# Single‐cell transcriptome analysis reveals evolving tumour microenvironment induced by immunochemotherapy in nasopharyngeal carcinoma

**DOI:** 10.1002/ctm2.70061

**Published:** 2024-10-16

**Authors:** Yaofei Jiang, Weixin Bei, Wangzhong Li, Ying Huang, Shuiqing He, Xiaobin Zhu, Lisheng Zheng, Weixiong Xia, Shuhui Dong, Qin Liu, Chuanrun Zhang, Shuhui Lv, Changqing Xie, Yanqun Xiang, Guoying Liu

**Affiliations:** ^1^ Department of Nasopharyngeal Carcinoma State Key Laboratory of Oncology in South China Guangdong Key Laboratory of Nasopharyngeal Carcinoma Diagnosis and Therapy Guangdong Provincial Clinical Research Center for Cancer Sun Yat‐Sen University Cancer Center Guangzhou China; ^2^ Department of Oncology the First Affiliated Hospital of NanChang University NanChang China; ^3^ Department of Thoracic Surgery and Oncology The First Affiliated Hospital of Guangzhou Medical University, State Key Laboratory of Respiratory Disease, National Clinical Research Center for Respiratory Disease, Guangzhou Institute of Respiratory Health Guangzhou China; ^4^ Department of Radiotherapy Sun Yat‐Sen University Cancer Center Guangzhou China; ^5^ Thoracic and GI Malignancies Branch National Cancer Institute, National Institutes of Health Bethesda USA; ^6^ Department of Pathology Guangdong Provincial People's Hospital (Guangdong Academy of Medical Sciences) Southern Medical University Guangzhou China; ^7^ Guangdong Provincial Key Laboratory of Malignant Tumor Epigenetics and Gene Regulation Department of Radiation Oncology Medical Research Center Sun Yat‐Sen Memorial Hospital, Sun Yat‐Sen University Guangzhou China

**Keywords:** immunochemotherapy effect, nasopharyngeal carcinoma, scRNA‐seq, tumour microenvironment

## Abstract

**Background:**

Combinatory therapeutic strategy containing immunochemotherapy as part of induction therapy components is one of the current trends in the treatment of high‐risk metastatic locally advanced nasopharyngeal carcinoma (NPC). However, the mechanism underlying the heterogeneity of response at the single‐cell level has not been underexplored.

**Methods:**

18 bulks and 11 single‐cell RNA sequencing from paired before‐treatment and on‐treatment samples in patients with treatment‐naive high‐risk metastatic locally advanced NPCs were obtained. Following quality control, a total of 87 191 cells were included in the subsequence bioinformatics analysis.

**Results:**

Immunochemotherapy was associated with on‐treatment tumour microenvironment (TME) remodelling, including upregulation of anti‐TMEs signatures, downregulation of pro‐TMEs signatures, reversing CD8^+^ T exhaustion, and repolarizing proinflammatory TAMs. For the patients achieving a complete response, the cytotoxic activity of CD8^+^ T cells was stimulated and more interferon‐gamma was provided, which would be the key for TAMs proinflammatory repolarization and eventually promote the CD8^+^ T cells maturation in turn. Among patients who did not reach complete response, differentiation and hypoxia signatures for endothelial cells were elevated after therapy. These patients exhibited higher levels of immune checkpoint genes in malignant cells at the baseline (before treatment), and decreased tumour antigen presentation activity, which may underlie the resistance mechanism to therapy.

**Conclusions:**

This study pictures a map of TME modulation following immunochemotherapy‐based combination induction therapy and provides potential future approaches.

**Highlights:**

- Immunochemotherapy remodeled T cell phenotypes.
- For the patients achieving complete response, more interferon gamma was provided by CD8^+^ T cells after therapy, which would be the key for TAMs pro‐inflammatory repolarization and eventually promote the CD8^+^ T cells maturation in turns.
- Among patients who did not reach complete response, malignant cells exhibited higher level of immune checkpoint genes before therapy, and decreased tumor antigen presentation activity, which may underlie the resistance mechanism to therapy.

## Graphical abstract

- Immunochemotherapy remodelled T‐cell phenotypes.
- For the patients achieving a complete response, more interferon‐gamma was provided by CD8^+^ T cells after therapy, which would be the key for TAMs pro‐inflammatory repolarization and eventually promote the CD8^+^ T cells maturation in turns.
- Among patients who did not reach a complete response, malignant cells exhibited higher levels of immune checkpoint genes before therapy, and decreased tumour antigen presentation activity, which may underlie the resistance mechanism to therapy.

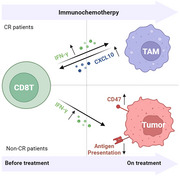

## 1 INTRODUCTION

Nasopharyngeal carcinoma (NPC) originates from the epithelium of nasopharynx mucosa and is characterized by strong invasiveness and high potential for metastasis.^1^ Over 75% of individuals are diagnosed with locally advanced NPC when their initial presentation.^2^ A standard treatment option for these patients is induction chemotherapy followed by concurrent chemoradiotherapy.^3^ For patients with stage IVA NPC, we have prospectively proven paclitaxel, cisplatin, and capecitabine (TPC) is superior to cisplatin and fluorouracil (PF) as induction treatment regimens.^4^ Recently, we confirmed the superiority of TPC chemotherapy compared with the standard regimen GP (gemcitabine and cisplatin) in advanced NPC in a prospective, multicenter, randomized controlled clinical study, which demonstrated better anti‐tumoral efficacy and favorable safety profile.^5^ However, patients with N3 disease, accounting for more than half of stage IVA NPC, still suffer from unsatisfactory prognoses, partially due to a lack of efficacious treatment strategies.

Immune checkpoint blockade has been demonstrated to be effective against advanced NPC in recent years. However, the combination of anti‐PD1 inhibitors with standard treatment as a curative setting in N3 locally advanced NPC did not show a significant survival benefit. However, several prospective trials have suggested the efficacy of anti‐EGFR, anti‐VEGF, and anti‐PD1 therapies in advanced NPC.^6^, ^7^, ^8^ A combination of these therapies could be a promising strategy to synergy and boost their anti‐tumour activities. Indeed, we prospectively conducted a phase II clinical trial (ChiCTR2000032317) evaluating the combination efficacy of anti‐PD1, antiangiogenic agent, and TPC chemotherapy in high‐risk NPC patients (TanyN3M0).^9^ Our result shows excellent distant metastatic control with acceptable safety for the combination therapy. However, a complex tumour microenvironment (TME) and its interactions with the combination therapies remain largely unexplored, which underscores the importance of decoding the complex mechanisms involved in such a treatment.

In the past, the illumination of immunomodulatory effects during the treatment has largely been performed with bulk RNA‐sequencing (RNA‐seq), which has low resolution and is unsuitable for examining cell‐type specific therapeutic responses.^10^ The emergence of the single‐cell RNA sequencing (scRNA‐seq) approach allows in‐depth insights into transcriptome characteristics at single‐cell resolution.^11^ Several studies have demonstrated the complex and dynamic TME landscape in NPC.^12^, ^13^, ^14^ Nevertheless, there is not currently a single‐cell study that allows for the rigorous analyses of changes in the TME with treatment and the association of these changes with clinical outcomes in NPC. Here, we performed scRNA‐seq of prospectively collected NPC specimens from this clinical trial to explore the therapy‐induced evolution of TME in NPC. A comprehensive understanding of how TME evolves and interacts longitudinally corresponding to therapy outcomes would help identify sensitivity and resistance mechanisms to current combined therapy and develop future novel immune‐modulating therapy strategies.

## 2 MATERIALS AND METHODS

### 2.1 Human specimens

This study was approved by the Sun Yat‐Sen University Cancer Center (SYSUCC) Institutional Review Boards and the Chinese Ethics Committee of Registering Clinical Trials (ChiCTR2000032317).^9^ The primary goal of the trial was to investigate the efficacy and safety of camrelizumab in combination with apatinib and induction chemotherapy in stage TanyN3M0 NPC. Forty‐nine patients pathologically diagnosed with NPC were enrolled. Paired before treatment (BT) and on treatment (OT) cervical metastatic lymph node samples were obtained using lymph node biopsy from the patients. The OT samples were obtained after one cycle of neoadjuvant therapy. The present study was an exploratory result of the clinical trial. We failed to get high‐quality paired OT specimens for one patient and finally, 11 lymph node samples from six patients were fresh‐processed for scRNA‐seq and another 18 lymph node samples from nine patients for bulk RNA‐seq. A written informed consent was obtained from each patient.

### 2.2 Sample preparation and scRNA‐seq

For each lymph node biopsy, at least two cylinders were collected; one was immediately processed to single‐cell dissociation on ice followed by scRNA‐seq and the other was soaked in formalin followed by standard histopathology. The library synthesis was conducted in accordance with the instructions of Chromium Next GEM Single Cell 3ʹ Reagent Kits v3.1 (10× GENOMICS). The cell concentration for each sample was standardized to 1000 cells/µL. Gel beads‐in‐emulsions (GEMs) were generated from cell suspensions using the chromium controller instrument. We isolated individual cells into droplets and coated gel beads with unique primers bearing unique cellular barcodes, unique molecular identifiers (UMIs), and poly(dT) sequences. Barcode‐labeled cells were mixed with reverse transcriptase, and GEM‐reverse transcription was performed in a Veriti 96‐well thermal cycler (Thermo Fisher Scientific). The reverse transcription was followed by amplification of the cDNA libraries using primers from the R1 and P5 arms. As part of the Chromium Single Cell Library v3 Kit, sequence‐ready libraries containing paired‐end constructs of Illumina were constructed. In brief, fragmentation, end repair, and A‐tailing of the cDNA library were performed, followed by ligation of the adapter after the right size was selected; finally, sample index PCR was performed and SPRI select beads were used for final purification. Finally, the barcoded sequencing libraries were 150 bp paired‐end sequencing on an Illumina Novaseq 6000 platform.

### 2.3 ScRNA‐seq data analysis

CellRanger software (10× GENOMICS) was used to generate row gene count matrices, which were then processed using the Seurat package (version 4.1.1) in R software (version 4.1.0).^15^ Cells with fewer than 201 or greater than 5999 unique genes, as well as those with a total UMI of fewer than 1001 or greater than 24 999, were excluded. Besides, cells with greater than 15% of mitochondrial gene counts were also removed. The gene count matrix for other cells was normalized and scaled with linear regression using the NormalizeData function and the ScaleData function, respectively. FindVariableFeatures function was used to select 2000 highly variable genes, which were used to identify major cell types. Then, principal component analysis (PCA) was used to reduce dimensionality for the genes, and the Uniform Manifold Approximation and Projection was used for dimension reduction for the 50 most informative principal components (PCs).^16^ Elbow plot was used to determine the number of used principal components. FindClusters function was used for cell clustering with default parameters. We annotated the major cell types by using the average expression of markers as follows: epithelial cells (EPCAM, KRT19, KRT18), T cells (CD3D, CD8A, CD8B, CD4), NK cells (FCGR3A, NKG7), endothelial cells (VWF, PECAM1), fibroblast cells (PDGFRB, COL1A1), B cells (CD79A, MS4A1, NEIL1), myeloid cell (LYZ, AIF1), mast cells (TPSB2, CPA3), plasma cells (MZB1). We used the DoubletFinder R package to predict doublets for data from each sample individually. Then, we removed predicted doublets with high confidence with a doublet rate of 3.3% in total. After doublets were removed, the Seurat R package was used to integrate the gene expression matrices of the 11 samples and transformed into a Seurat object. When data scaling, “S.Score” and “G2M.Score” were calculated using the CellCycleScoring function, and the percent of mitochondrial counts calculated by the PercentageFeatureSet function was used.^17^ The confounding factors were eliminated using the Harmony package (version 1.0) based on the top 20 PCA components identified.^18^ Similar procedures including normalization, dimension reduction, cell integration with harmony, and clustering were performed for subclustering analysis within the major cell types (T cells, myeloid cells, and endothelial cells). The FindClusters function resolution parameters were disparate among different cell types as follows: .5 for all cells and myeloid cells, 1 for T cells, and .2 for endothelial cells. FindAllMarkers function with default two‐sided non‐parametric Wilcoxon rank sum test was used to identify different expression genes (DEGs) for each sub‐cluster. The DEGs of each subcluster and the previously reported cellular markers were used to annotate the cell groups.

### 2.4 Differential expression and function gene set analysis

To further investigate the functional and mechanistic insights of clusters or specific groups. The differential over‐expressed genes between specific groups were identified using the FindMarkers function using default test, and the group‐specific overrepresented Gene Ontology (GO) and KEGG Pathway enrichment analyses were calculated by the clusterProfiler package (version 4.0).^19^ Gene set enrichment analysis (GSEA, version 1.56.0) using sort DEGs identified above between specific groups was used to find the pathways that enriched in the corresponding groups. Further, gene set variation analysis (GSVA, version 1.42.0) with 50 hallmark gene sets in the MSigDB databases in specific groups was also applied,^20^ with 29 hallmark gene sets described for tumour microenvironment classification in all cells,^21^ and 14 tumour‐relative hallmark pathways in malignant cells. To explore the interested potential function of cell groups, AddModuleScore function in the Seurat package was used to compute the module/signature enrichment scores for each cell. All gene signature data sets were collated from previous relevant literature or MSigDB databases, and the involved genes of each signature were summarized in Table S4.

### 2.5 Trajectory inference analysis

The Monocle 2 package (version 2.22.0) was applied to define pseudotime trajectories of CD8^+^ T cells, CD4^+^ T cells, and tumour‐associated macrophage cells.^22^ Input files were gene‐cell matrices in the scale of raw UMI counts extracted from former Seurat data. Then, the newCellDataSet function was used to create a monocle object. The variable differential genes with a min expression greater than .5 between cell clusters were identified and potentially involved in the trajectory analysis. After that, the reduceDimension function was used for dimension reduction with the “DDRTree” method and “max_components” of 2. Plot_cell_trajectory function was used to order and visualize cells and then differentialGeneTest function was used to calculate gene changes along with the pseudotime. The top 500 genes with the lowest *q*‐value were clustered into subgroups based on their expression patterns. For each subgroup, the GO biological process was calculated using the clusterProfilter package described above.

### 2.6 Single‐cell copy number analysis

Malignant cells were identified from nonmalignant epithelial cells by calculating copy number variation (CNV) in previously identified epithelial cells using the inferCNV (version 1.10.1) package (https://github.com/broadinstitute/inferCNV). Fibroblast cells were used as a reference, and sex chromosomes were excluded data. To distinguish malignant in the present study, we performed K‐means clustering analysis separately on epithelial cells and the reference cells derived from each sample according to CNV scores. Nonmalignant cell clusters were identified as those mainly containing fibroblast cells. On the contrary, malignant cells were regarded as those with high CNV scores in other clusters.

### 2.7 SCENIC analysis

The SCENIC package (version 1.2.4) was used for the gene regulatory network.^23^ TFs motif enrichment and co‐expression modules analysis from the data supported the procession. Regulon specificity score in the AUCell package (version 3.12) was used to calculate and rank the activities of TFs.^24^

### 2.8 Cellular interaction analysis

We performed cell–cell communication analysis using CellChat (version 1.1.3) to explore potential cellular interactions among cell subtypes in the NPC TME, which has built receptors and ligands and their communications for humans in the CellChat database.^25^ We evaluated the specific interactions in CR responder BT, CR responder OT, PR responder BT, and PR responder OT. Then, differential signalling changes were assessed between (1) BT versus OT, and (2) CR responder BT versus non‐CR responder BT.

### 2.9 RNA‐sequencing data analysis

Eighteen lymph‐node biopsied specimens from nine patients (including nine BT and nine matched OT samples) were proceeded with RNA sequencing. Trizol (Invitrogen) was used to extract the total RNA, and then NanoDrop and Agilent 2100 bioanalyzer (Thermo Fisher Scientific) was used to qualify and quantify the total RNA. mRNA library was amplified and then sequencing data was filtered with SOAPnuke (v1.5.2).^26^ HISAT2 (v2.0.4) was used to map clean reads to the reference genome (GCF_000001405.39_GRCh38.p13).^27^ Bowtie2 (v2.2.5)^28^ was applied to align the clean reads to the reference coding gene set, and then the expression level of genes was calculated by RSEM (v1.2.12).^29^

### 2.10 Exploration of public datasets

To further validate the features variations of tumour microenvironment on the prognosis of NPCs, including cell compositions, gene regulation, and functional alteration, we collected bulk RNA‐seq data publicly available for NPCs from the Gene Expression Omnibus (GEO, GSE102349 dataset which contains 88 patients having prognosis data [https://www.ncbi.nlm.nih.gov/geo/]).^30^

### 2.11 Multiplex immunohistochemistry

Multiplex immunohistochemistry (IHC) staining method was performed to visualize the expression of CD8, PD‐1, FOXP3, CD68, CK in the TME of BT and OT lymph node biopsy, using the PANO 7‐plex IHC kit, cat 0004100100 (Panovue) following mature instructions. Fresh biopsied specimens were stabilized using formalin, and dehydration and paraffin embedding were performed. 5 µm‐thick sections were cut from paraffin blocks for slide making. The slides were then processed for the subsequent multiplex IHC staining assays. In brief, following primary antibody application, a horseradish peroxidase‐conjugated secondary antibody was used and tyramide signal amplification (TSA) was performed. Using microwave treatment after each TSA operation, nuclei were stained with 4′‐6′‐diamidino‐2‐phenylindole (SIGMA‐ALDRICH). Primary antibodies included anti‐CD8A (mouse; CST; cat. no. CST70306; 1:100), anti‐PD1 (rabbit; CST; cat. no. CST86163; 1:100), anti‐FOXP3 (mouse; Biolegend; cat. no. BLG320202; 1:50), anti‐CD68 (rabbit; CST; cat. no. CST76437; 1:1000), and anti‐CK (mouse; SIGMA; Cat. No. C2562; 1:300). Whole‐slide scanning fluorescent images was performed using an Olympus VS200 (Olympus Germany). Whole slide fluorescence images were analyzed with QuPath software.^31^

### 2.12 Cell lines

Human THP‐1 mono‐cytes (# CL‐0233; Pricilla) were cultured in RPMI‐1640 (Gibco BRL) supplemented with 10% fetal bovine serum (ExCell Bio) and 1% penicillin/streptomycin (Invitrogen). All cells were incubated with 5% CO_2_ at 37°C in a humidified incubator. To induce M0‐type Mφs, about 1 × 10^6^ THP‐1 monocytes were stimulated with phorbol 12‐myristate 13‐acetate (185 ng/mL, Sigma) for 6 h.

### 2.13 Flow cytometry assay

Harvested Mφs and CD8^+^ T cells were made into single‐cell suspensions by trypsin digestion. After washing, Mφs and CD8^+^ T cells were stained with CD163 (#333607, Biolegend), CD206 (#321109, Biolegend) PD‐1 (#329920, Biolegend), CXCR3 (#353742, Biolegend) on ice away from light according to the manufacturer's instructions.

### 2.14 Immunohistochemistry staining

IHC staining was performed using a Polymer HRP IHC kit (#RS0050, Immunoway). Briefly, 4‐µm thick tissue sections of formalin‐fixed, paraffin‐embedded NPC tissues were deparaffinized in xylene and rehydrated through graded alcohol. Then, antigenic retrieval was performed with sodium citrate and a high‐pressure boiler for 20 min. After cooling to room temperature, the sections were incubated with 3% hydrogen peroxide for 10 min to inhibit endogenous peroxidases. Primary antibodies against CD68 (1:400 dilution, #DF7518, Affinity), LYZ (1:400 dilution, #66456‐1‐lg, proteintech), and MS4A6A (1:200 dilution, #PA5‐72732, Thermo Fisher Scientific) were incubated at 4°C overnight. The results of IHC staining were observed and imaged under a light microscope (Olympus).

### 2.15 ELISA

Elisa was performed using GZMK and GZMA Elisa kit (#SEB209Hu and SEA599Hu, Cloud‐Clone Corp). Collect plasma using EDTA or heparin as an anticoagulant. Prepare seven wells for standard and one well for blank. Add 100 µL each of the samples into the appropriate wells. Cover with the plate sealer. Add 100 µL of detection reagent A working solution to each well and cover the wells with the plate sealer. Aspirate the solution and wash with 350 µL of 1× wash solution to each well using a squirt bottle, multi‐channel pipette, manifold dispenser, or autowasher, and let it sit for 1∼2 min. Invert the plate and blot it against absorbent paper. Add 100 µL of detection reagent B working solution to each well, and cover the wells with the plate sealer. Add 90 µL of substrate solution to each well. Cover with a new Plate sealer. Protect from light. Add 50 µL of stop solution to each well. The liquid will turn yellow with the addition of the Stop solution. Remove any drop of water and fingerprint on the bottom of the plate and confirm there is no bubble on the surface of the liquid. Then, run the microplate reader and conduct measurement at 450 nm immediately.

### 2.16 Statistics analysis

We used R (version 4.1.0) for all statistical analyses. Group differences were analyzed using chi‐square, Student's *t*, paired *t*, Wilcoxon, Pearson correlation, and Kruskal–Wallis tests, as appropriate. We analyzed the survival data using the Kaplan–Meier method and examined the relationships between the groups using Spearman's correlation analysis. The optimal cutoff value was used to define the gene expression levels for survival analyses in this study. A *p*‐value of .05 was regarded as statistically significant for all tests.

## 3 RESULTS

### 3.1 Landscape of single‐cell profiling before and on treatment

**FIGURE 1:**
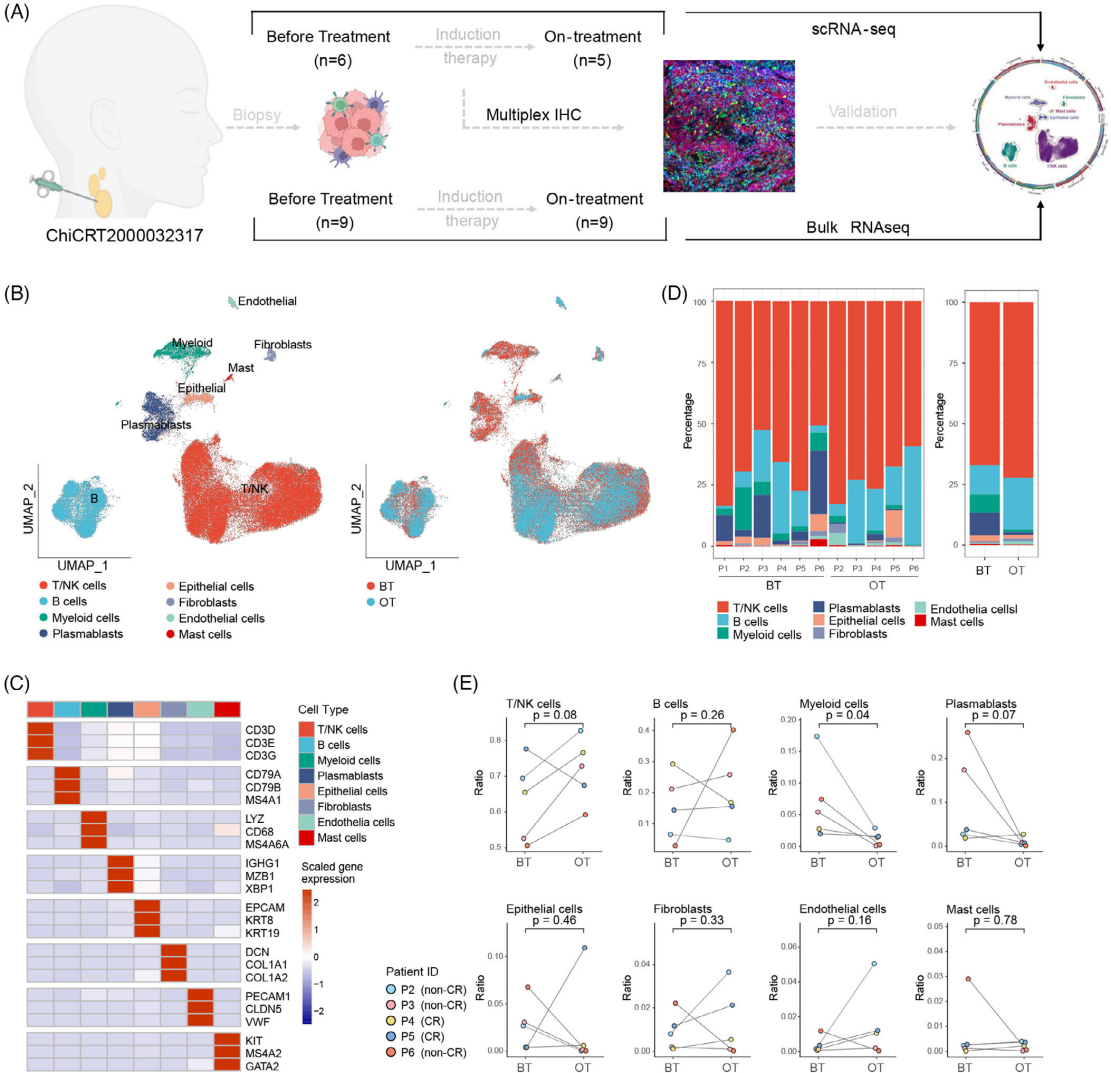
Study overview and landscape of tumour microenvironment of NPC during treatment. (A) Scheme of the study design. We obtained fresh needle‐biopsied samples for single‐cell sequencing from 11 paired metastatic cervical lymph nodes of six patients with nasopharyngeal carcinoma (NPC) before and after one cycle of neoadjuvant therapy. Another 18 paired cervical lymph node biopsied samples were obtained for bulk RNA sequencing. (B) Uniform manifold approximation and projection (UMAP) plots of all high‐quality cells coloured by eight major cell types and sample types. (C) Heatmap of cell‐type‐defining genes between each major cell type. Solid colours from dark blue to dark red represent scaled gene expression levels from low to high. (D) Bar plots of cell‐type proportions grouped by sample source and sample type. (E) Proportions of the eight major cell types in paired before‐/on‐treatment samples. The significance of differential proportion (*p*‐value) was determined by paired *t*‐test. The samples were named as following example, “BT2” and “OT2” representing before and on treatment metastatic cervical lymph nodes of patient P2, respectively. IHC, immunohistochemistry; BT, before treatment; OT, on treatment; CR, complete response.

To explore the combination therapy‐induced changes of TME in NPC, we used scRNA‐seq to profile 11 fresh needle‐biopsied samples obtained from 6 individual patients enrolled in trial ChiCTR2000032317 (Figure 1A). Samples were categorized by two time points (before treatment, BT; on treatment, OT) and subgrouped by the response outcome to induction therapy [complete response (CR) and non‐CR], respectively. The detail of patient information is displayed in Table S1. After quality control, 87 191 cells were included in the following analysis (Figure S1A–E, Table S2). Eight major cell subtypes were identified using classical markers (Figure 1B, C). These cell clusters were widespread among samples, but noticeable discrepancies in cell composition were evidenced among samples and between different biopsy time points within the same patient (Figure 1D, Table S2). There was a significant decrease in myeloid cell population in OT samples compared with BT samples (*p* = .04, Figure 1E), but no significant differences were observed in other cell types. Besides, we observed a rising trend of T/NK cell ratio in the majority of OT samples (Figure 1E and Figure S2A). In the meantime, we inferred the constitute of eight major cell types in above‐mentioned bulk RNA‐seq samples using the CIBERSORTx. We found that B cells and T/NK cells were increased significantly in OT samples while epithelial cells and fibroblast cells decreased significantly (Figure S2B). Notably, patients with abundant epithelial cells and endothelial cells in the GSE102349 dataset exhibited a worse progression‐free survival (PFS, Figure S2C). These changes suggest there is an underlying TME landscape transformation process responding to the combination therapy in these NPC patients.

### 3.2 Therapy‐induced plasticity of TME

**FIGURE 2:**
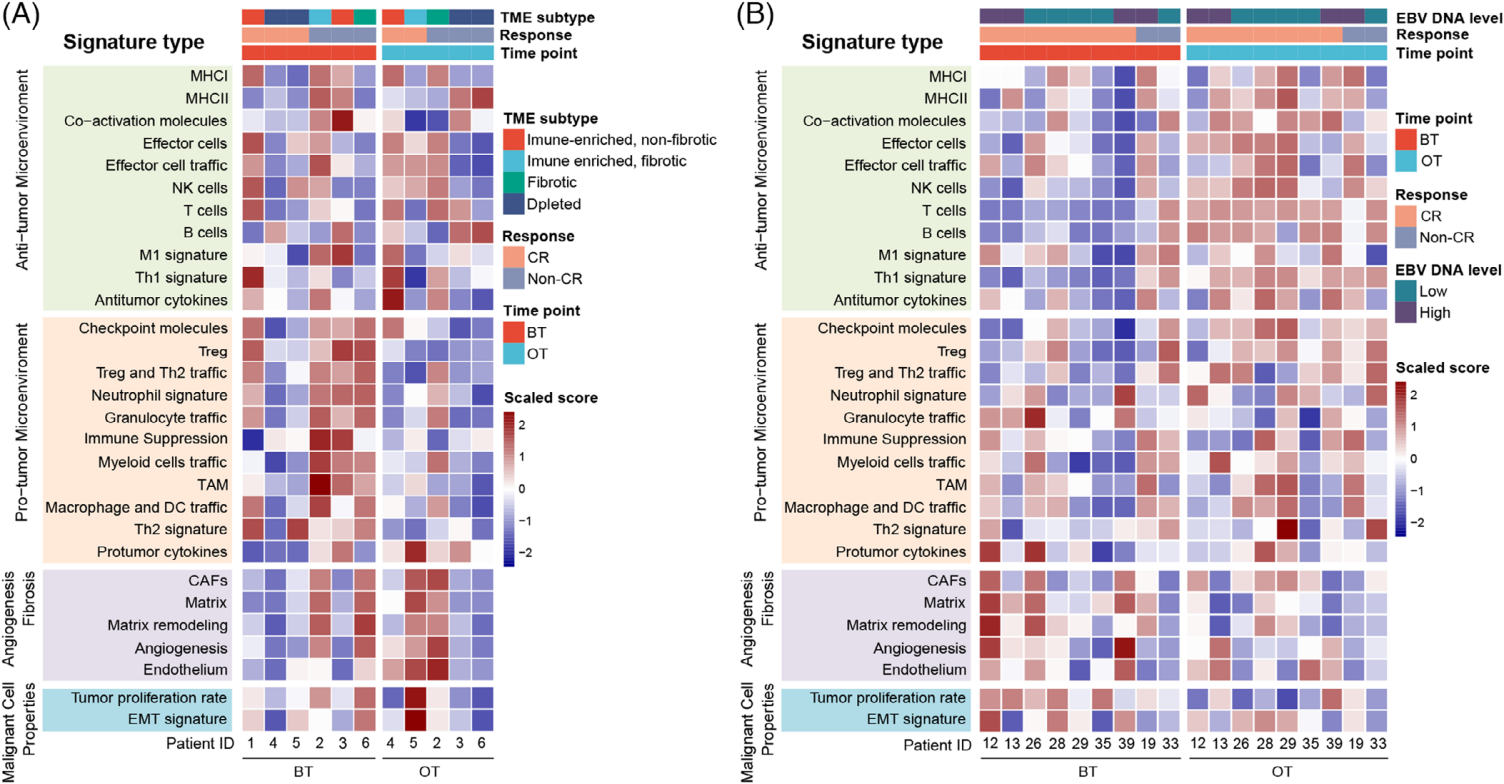
Immunochemotherapy‐based treatment remodels the TME in NPC. Heatmap of pan‐cancer TME signatures GSVA scores across (A) 11 paired single‐cell sequencing samples (BT [*n* = 6] and OT [*n* = 5]). (B) 18 paired bulk RNA‐seq samples (BT (*n* = 9) and OT (*n* = 9)). Solid colours from dark blue to dark red represent scaled signature score from low to high.

Subsequently, we employed widely acknowledged pan‐cancer TME signatures to assess the immune components within the TME and investigate the potential for combined therapy to remodel TME, taking into account the variability observed among different responders.^21^ We classified each sample into four distinct microenvironment subtypes accordingly (Figure 2A). We observed distinguishing TME landscape patterns between BT and OT samples, as well as between CR and non‐CR patients. Interestingly, signatures related to pro‐TME were widely suppressed across the OT samples, indicating the inhibition of the pro‐tumoral machinery by the combination therapy (Figure 2A). In the meantime, we performed bulk RNA sequencing (RNA‐seq) of 18 paired lymph node specimens biopsied from nine patients to verify these findings further. The expression of anti‐TME signatures in OT samples revealed by bulk RNA‐seq analysis was upregulated, as shown in Figure 2B. Moreover, BT samples from CR patients exhibited lower pro‐TME signatures compared with their non‐CR counterparts, including tumour‐associated macrophage (TAM) signatures, myeloid cell traffic signatures, pro‐tumour cytokine signatures, and signatures of immune suppression. These observed outcomes collectively suggest that the combined therapy appears to promote remodelling of the NPC TME. In addition, the baseline pro‐TME signatures may serve as predictors for predicting responses to the combination treatment.

### 3.3 Temporal dynamics of T‐cell subsets and association with response

**FIGURE 3:**
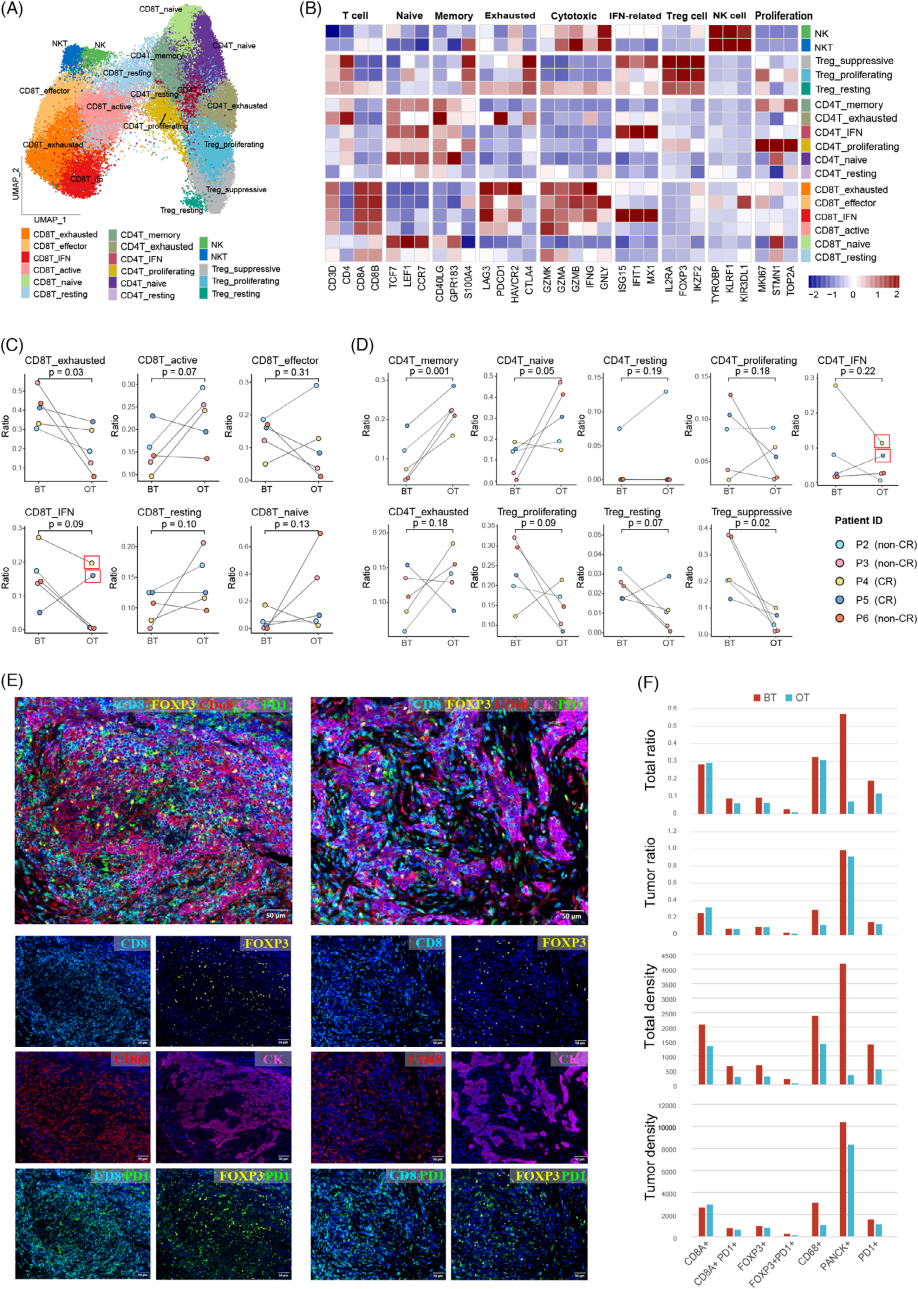
T cells are differentially remodelled by therapy. (A) UMAP map of T/natural killer (NK) cells, as indicated by the colours and labels. (B) Heatmap of mean expression of canonical functional markers in each cell cluster, including T‐cell markers, naïve markers, memory markers, exhausted markers, cytotoxic markers, interferon‐related (IFN‐related) markers, Treg cell markers, NK cell markers, and proliferation markers. Solid colours from dark blue to dark red represent scaled gene expression levels from low to high. (C) Corresponding change of CD8^+^ T‐cell subtypes proportion from individual patients in paired BT (*n* = 5) and OT (*n* = 5) samples. (D) Corresponding change of CD4^+^ T and Treg cell subtypes proportion in paired BT (*n* = 5) and OT (*n* = 5) samples. Significance of differential proportion (*p*‐value) was determined by paired t‐test. (E) Multiplex immunohistochemistry images characterizing the TME of NPC before (left) and on treatment (right) in a representative case (P4) who showed CR response to one cycle of the combination induce therapy. (F) Histogram showing the quantitative ratio and density of TME cells between BT and OT samples in tumour and all the image areas.

A total of 60389 T/NK cells were re‐clustered. We identified 17 T‐cell subtypes, based on the combination of cluster‐specific DEGs (Table S3, Figure S2D) and canonical functional markers (Figure 3A, B), which included six CD4^+^ T‐cell clusters, six CD8+ T‐cell clusters, three Treg clusters, one NK cell cluster, and one NKT cell cluster. We observed that the CR patients exhibited a lower level of Treg cells and a higher proposition of CD8^+^ T cells at baseline (before treatment), compared with non‐CR patients (Figure S2E). Furthermore, we examined the expression of naive, cytotoxic, and exhausted signatures for each cluster (Figure S2F). We observed that cytotoxic markers were widely upregulated in NK cells, CD8^+^ T cells, and NKT cells, which corresponded to their specific anti‐tumour effects (Figure 3B). Besides, exhausted markers were also upregulated across CD8^+^ T cells, which was consistent with previous reports.^14^, ^32^

We next examined the changes of T/NK cell subclusters in available matched BT and OT samples (Figure 3C, D). The proportion of CD8T_exhausted cells decreased significantly in OT samples compared with BT samples (*p* = .03, Figure 3C). Notably, one of the patients with negative plasma EBV DNA at the baseline (P5) showed decreased CD8T_IFN cells on the treatment with induction therapy, which was different from other plasma EBV DNA positive samples, which may suggests that EBV DNA negative NPC may exhibit a distinct response mechanism, which is different from EBV DNA positive NPC (Figure 3C). There was a significantly increased proportion of CD4T_memory cells (*p* = .001) and significantly decreased Treg_suppressive cells in OT samples (*p* = .015) (Figure 3D). Besides, compared with BT samples, OT samples tended to have a higher proportion of CD4T_naive cells, lower levels of Treg_proliferating, and Treg_ resting cells (Figure 3D). Notably, in OT samples, CR patients had a higher proportion of CD8T_IFN cells and CD4T_IFN cells compared with other non‐CR patients (Figure 3C, D).

To verify the findings above, a multiplex immunohistochemistry assay was performed using corresponding BT and OT samples in a representative case (P4, Figure 3E, F). The results showed that the ratio and density of CD8^+^ T cells (CD8A+) in the tumour area increased in the OT sample. The ratio and density of the CD8T_exhausted cells (CD8A+PD1+), Treg cells (FOXP3+), Treg_suppressive cells (FOXP3+PD1+), and malignant cells (CK+) in both total area and tumour area decreased in the OT sample. Also, PD1+ cells decreased in both ratio and density. Besides, the proportion of myeloid cells also decreased after therapy, with the expression of CD68, LYZ, and MS4A6A decreased, which were usually used together as markers of myeloid cells (Figure 3E, F, Figure S3). All these results suggested that the immune microenvironment was repolarized to benefit from immune checkpoint inhibitor (ICB) combined therapy.

### 3.4 Changes in T‐cell transcriptions associated with treatment and response

**FIGURE 4:**
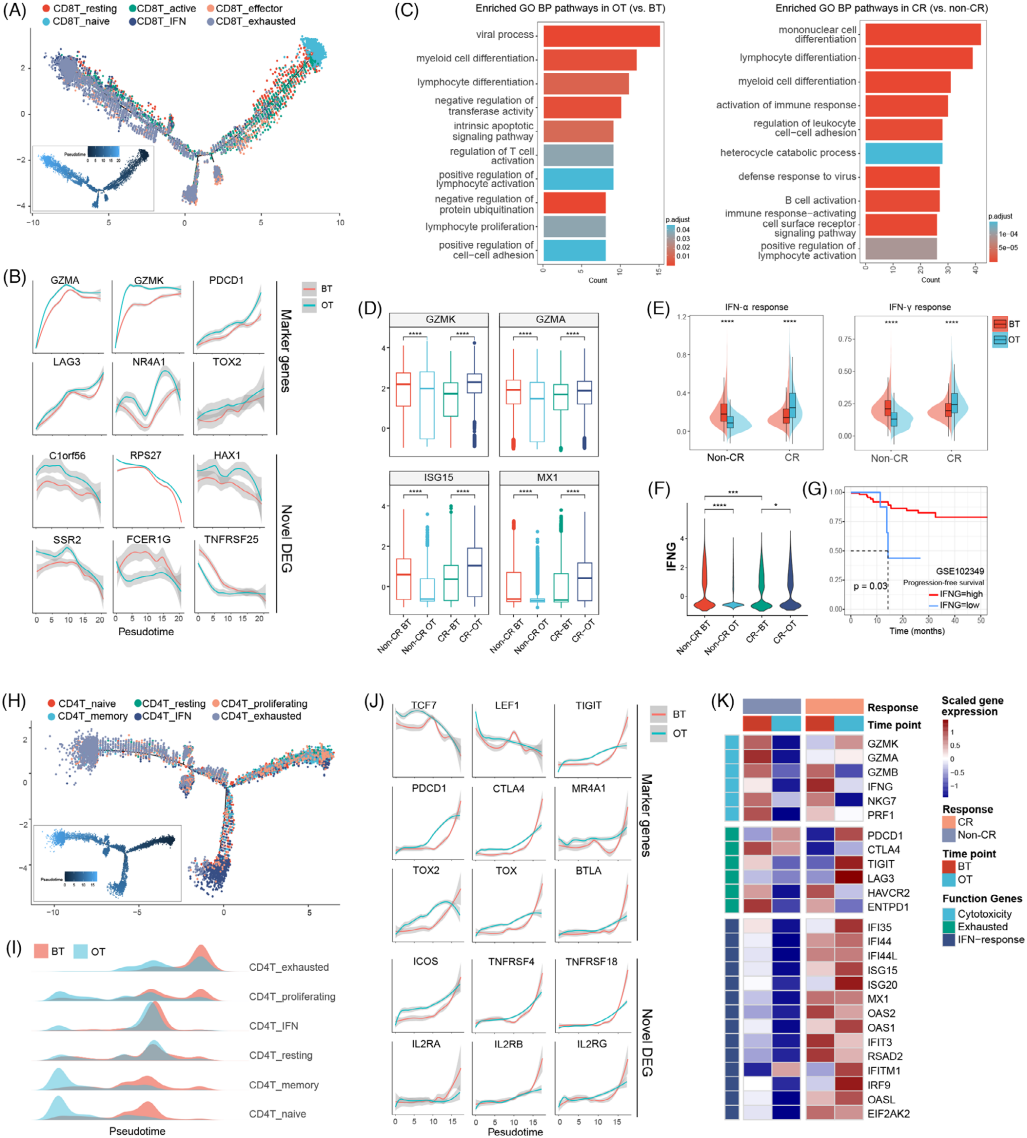
Differentially expressed genes in CD8^+^ and CD4^+^ T cells. (A) Pseudotime trajectories for CD8^+^ T cells inferred by Monocle 2. Each point corresponds to one single cell and each colour represents one CD8^+^ T‐cell subgroup. Each cell was scaled by pseudotime scores and coloured using default colour (inlet panel). (B) Plot of marker and functional genes along the CD8^+^ T‐cell trajectory distinguished by BT and OT samples. (C) Gene Ontology (GO) biological process (BP) analysis of CD8^+^ T cells upregulated DEGs in OT samples compared with BT samples (left panel), and CD8^+^ T cells upregulated DEGs in CR patients compared with non‐CR patients in BT samples (right panel). (D) The relative RNA expression of representative genes using scRNA‐seq of BT and OT samples from CR and non‐CR patients in all CD8^+^ T cells. (E) Signature score distributions for IFN‐α response and IFN‐γ response signatures within CD8^+^ T cells. (F) Violin plots showing the relative RNA expression of IFN‐γ (IFNG) using scRNA‐seq of BT and OT samples from CR and non‐CR patients. (G) Progression‐free survival curves of the patients with NPC from the GSE102349 dataset, stratified by the IFNG expression. The red line shows the survival curve of the patients contain higher IFNG expression, while the blue line shows the survival curve of the remaining patients. (H) Pseudotime trajectories for CD4^+^ T cells inferred by Monocle 2. Each point corresponds to one single cell and each colour represents one CD4^+^ cell subgroup. Each cell was scaled by pseudotime scores and coloured using default colour (inlet panel). (I) The cell density distribution of the pseudotime‐ordered CD4^+^ T subtypes distinguished by BT and OT samples. (J) Plot of marker and functional genes along the CD4^+^ T‐cell trajectory. (K) Heatmap shows the normalized mean expression of representative genes using scRNA‐seq of BT and OT samples from CR and non‐CR patients. Solid colours from dark blue to dark red represent scaled gene expression levels from low to high. In D, E, and F, *p*‐values were estimated by *t*‐test. ns: not significant. *p < .05, **p < .01, ***p < .001, ****p < .0001. Gray shading in (B), and (J) represents the 95% confidence interval at any given pseudotime.

Next, we generated pseudotime trajectories using Monocle 2 to explore changes in the T‐cell development. CD8T_naïve cells with high expression of CCR7 and LEF1 were considered as the root of the trajectories. Consistent with a recent study,^14^ three main statuses were observed along trajectories that developed from CD8T_naïve cells in the beginning, then branched into CD8T_active cells and CD8T_effector cells in the middle, and to CD8T_exhausted cells in the terminal point (Figure 4A). Consistent with the proportion analysis above, CD8T_exhausted cells were more frequent at the end of the trajectory in BT samples. Besides, we observed that cytotoxic markers (GZMK, GZMA), as well as exhausted related genes (LAG3, PDCD1, NR4A1, and TOX2), were higher continually in OT samples (Figure 4B). Some DEGs were also found consistently higher (C1orf56, RPS27, HAX1, SSR2) or lower (FCER1G, TNFRSF25) in OT samples which would be related to the expression of immune checkpoints, like PDCD1 and TOX2 (Figure 4B). We performed GO BP enrichment analyses and found the enrichment in myeloid cell differentiation and positive regulation of lymphocyte activation in OT samples versus BT samples, as well as in CR patients versus non‐CR samples (Figure 4C). This indicated that the combination therapy activates lymphocyte activity and enhances myeloid cell differentiation, which reaches a higher degree in patients with CR.

Earlier research has demonstrated the presence of an exhausted progenitor population within infiltrating CD8^+^ T cells. These cells have been linked to a more favourable response to ICB therapy.^33^, ^34^ We observed that OT samples had higher progenitor signature scores in CD8T_exhausted cells. This indicates that the CD8T_exhausted cells tend to adopt an anti‐tumour phenotype (Figure S4A). Also, NPC patients with a high score of progenitor signature exhibited a longer PFS in our cohort (*p* = .007 and *p* < .001, respectively, Figure S4B). Additionally, when performing pathway enrichment analysis on CD8T_exhausted cells using KEGG, a distinct pattern was shown between BT and OT samples. In BT samples, there was an upregulation in apoptosis, the p53 signalling pathway, and the platinum drug resistance pathway. Conversely, OT samples exhibited an upregulation in the antigen processing and presentation pathway. This further validates the phenotype transformation of CD8^+^ T cells (Figure S4C). We next examined function gene expression differences based on time points and response. Interestingly, both the representative cytotoxicity genes (GZMK, GZMA) and IFN response‐related genes (ISIG15, MX1) were upregulated significantly in CD8^+^ T cells after therapy in CR patients but downregulated in non‐CR patients (Figure 4D). Furthermore, the expression of GZMK and GZMA in the serum of these patients determined by ELISA assay confirmed that GZMK and GZMA upregulated only in CR patients (Figure S4D). IFN‐α/γ response signatures consistently hoisted only in CR patients (Figure 4E). In addition, the expression of IFNG was significantly higher in CR patients compared with those with non‐CR at the baseline (before treatment), which was strongly related to longer PFS, and it decreased only in non‐CR patients (Figure 4F, G).

In parallel, we used Monocle 2 to analyze a trajectory in the CD4^+^ T‐cell population, which revealed that the cells started in CD4T_naïve cells, progressed through CD4T_memory cells, then branched into CD4T_IFN cells, followed by CD4 T_proliferating cells and CD4T_resting cells, and eventually developed into CD4T_exhausted cells at the end of trajectory (Figure 4H). In the meantime, we observed more frequent CD4T_naive cells and CD4T_memory cells at the initial point in OT samples versus BT samples (Figure 4I). Naïve (TCF7, LEF1), exhausted (TIGIT, PDCD1, CTLA4, TOX2), and IL2R‐related genes (IL2RA, IL2RB, IL2RG) increased in OT samples, but decreased at the end of the trajectory where CD4T_exhausted cells existed (Figure 4J). Consistent with CD8^+^ T cells, IFN‐related genes are generally upregulated in both BT and OT samples in CR patients compared with those with non‐CR (Figure 4K). Interestingly, three potential differentiation states were observed along the trajectory path, that OT samples had more CD4^+^ T cells gathered at the beginning of the trajectory (state 1) and less at the terminal state (state 3) (Figure S4E). The radiation heatmap showed cell state 2 exhibited more differential genes associated with activation of the innate immune response, defence response to viruses, and interferon‐mediated signalling pathway, while cell state 3 had more genes related to the regulation of cell cycle phase transition, regulation of T‐cell activation, and cellular respiration (Figure S4F).

We next explored the dynamic transcriptomic change of Treg cells, which usually play a role in immune suppression. GSEA analysis demonstrated downregulated pathways associated with adverse prognosis, e.g., p53 pathway, JAK‐STAT pathway, and apoptosis, which indicates the transition of Treg cell status upon the combination therapy (Figure S4G). We observed the highest expression levels of IL2R, inhibitory, and co‐stimulatory related genes in the Treg_suppressive subtype (Figure S4H).^14^ Pathway enrichment analysis revealed that cytokine‐mediated pathway, NF‐kB pathway, and TNF signalling pathway were highly enriched in Treg_suppressive cells, consistent with their immune suppressive phenotype mentioned above (Figure S4I, J). The ratio of Treg_suppressive cells inferred in the 18 paired bulk RNA‐seq samples decreased significantly after therapy (*p* = .002, Figure S4K), which is consistent with the observation from scRNA‐seq analysis (Figure S2E) and is associated with shorter PFS (*p* = .005, Figure S4L).

The observations above manifest that combination therapy may enhance the development of an anti‐tumour microenvironment in NPC by remodelling the composition and function of T cells. The baseline TME composition and its alterations vary among patients, potentially reflecting differences in their response to treatment efficiency.

### 3.5 Transformation of tumour‐associated macrophage cells

**FIGURE 5:**
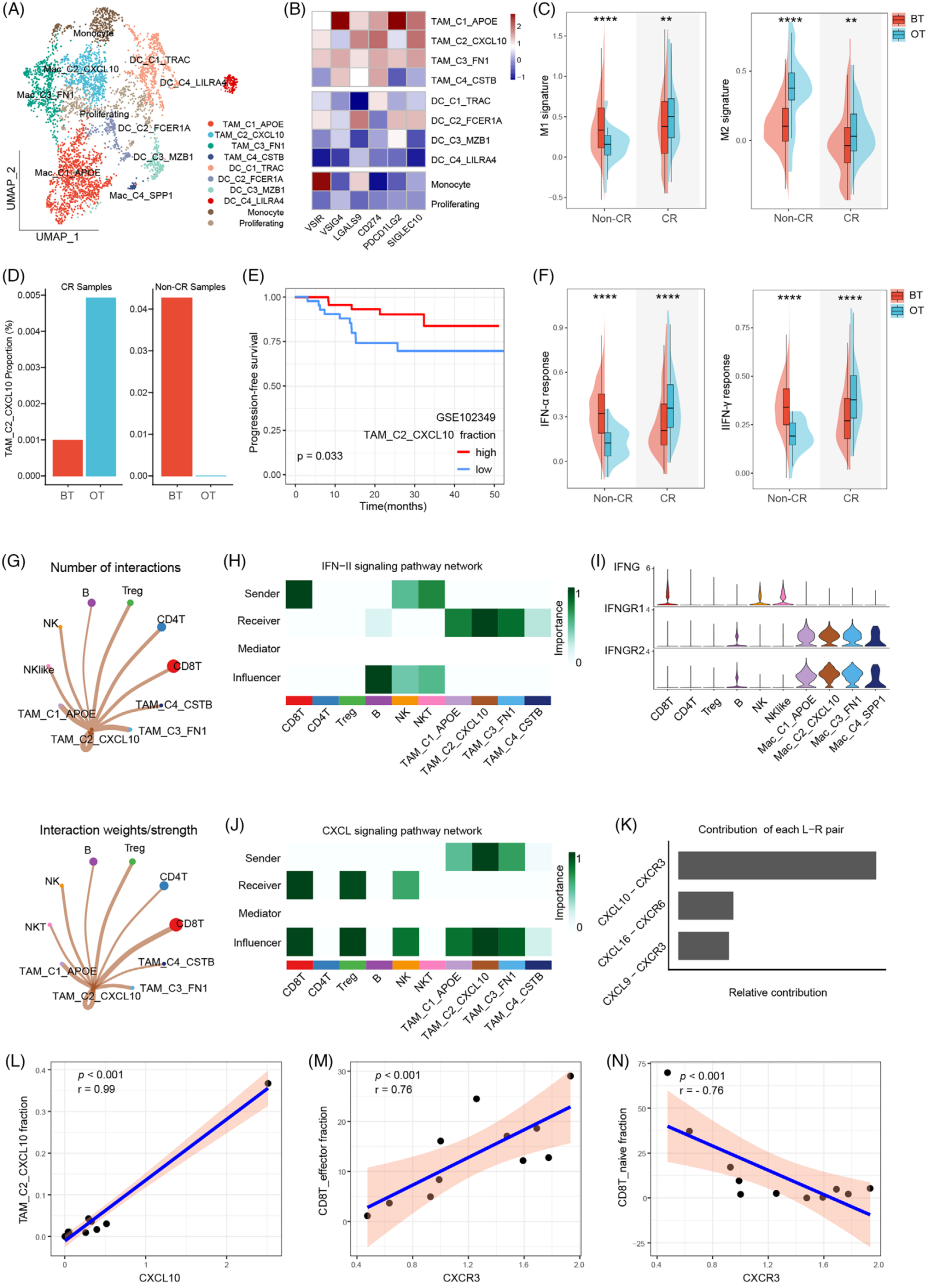
Tumour‐associated macrophages pro‐inflammatory repolarization and interacted with CD8^+^ T cells. (A) UMAP of myeloid sub‐cluster cells, as indicated by the colours and labels. (B) Heatmap shows the normalized mean expression of immunomodulatory genes across all myeloid cell types. Filled colours from dark blue to dark red represent scaled expression levels from low to high. (C) Signature score distributions for M1 and M2 signatures within TAM cells. (D) The histography showing the proportion of TAM_C2_CXCL10 normalized by all myeloid cells in BT and OT samples. (E) Progression‐free survival curves of the patients with NPC from the GSE102349 dataset, stratified by the fraction of TAM_C2_CXCL10 cells which were inferred by CIBERSORTx. The red line shows the survival curve of the patients contain high cell fraction, while the blue line shows the survival curve of the remaining patients. (F) Signature score distributions for IFN‐α response and IFN‐γ response signatures within TAM cells. (G) Circle plot shows the interaction number and strength of interactions among the TAMling subtypes and other cells. (H) The role of TAMs subtypes and other cells in the IFN‐II signalling pathways. (I) Violin plot shows the expression of IFN‐II signalling pathway genes across TAM subtypes and other cells. (J) The role of TAMs subtypes and other cells in the CXCL signalling pathways. (K) The relative contribution of receptor–ligand pairs in the CXCL signalling pathways between TAM subtypes and other cells. (L) Plot of correlation between CXCL10 expression and fraction of TAM_C2_CXCL10 cells normalized by myeloid cells. (M) Plot of correlation between CXCR3 expression and a fraction of CD8T_effector cells normalized by CD8^+^ T cells. (N) The plot of correlation between CXCL10 expression and fraction of CD8T_naive cells normalized by CD8^+^ T cells. In C and F, the significance of differential signature enrichment (*p*‐value) between samples was determined by t‐test. ns: not significant. *p < .05, **p < .01, ***p < .001, ****p < .0001.

The myeloid cell population, especially macrophages, significantly decreased after treatment (Figures 1E and 3E, F). We re‐clustered myeloid cells into 10 clusters (Figure 5A and Figure S5A–C, Table S2–3). Immunomodulatory genes were commonly expressed across various myeloid subpopulations, with particularly notable expression levels observed in TAMs (Figure 5B).^35^, ^36^ Significantly, VSIR, VSIR4, and LGALS9, which product proteins that promote T‐cell immune checkpoint, and SLGLEC10, which inhibits inflammation, were also upregulated in TAMs from OT samples versus BT samples (Figure S5D).^37^, ^38^, ^39^, ^40^ These phenomena were similar to the results observed in renal cell carcinoma in a recent study.^41^ Meanwhile, DEG analysis showed upregulation of antigen cross‐presentation (HLA genes) and chemotactic factor (CXCL11) in OT samples (Figure S5E). Also, antigen cross‐presentation‐related genes upregulated in CR patients in BT samples compared with those non‐CR patients (Figure S5F). These genes are crucial for the regulation of the immune system, influencing its development, homeostasis, and functionality.^42^ Intriguingly, SPP1, which is highly related to the evolution of malignant cells and reprogramming of microenvironment, and poor treatment outcomes from the combination therapy,^43^, ^44^ was heightened expressed in BT samples and non‐CR patients (Figure S5E,F). Patients with high expression of SPP1 exhibited a shorter PFS (Figure S5G). It is undecided if SPP1 in TAMs plays a critical role in our study.

Numerous studies have shown that relying solely on a basic classification of M1/2 phenotypes for macrophages is insufficient to fully grasp the internal variability and diversity of TAM phenotypes.^45^, ^46^ We assessed the M1 and M2 signatures of all TAMs and observed that the TAM‐C1‐APOE population exhibited the highest M2 signature score, indicating a polarization towards the M2 phenotype. Conversely, the TAM_C2_CXCL10 population showed the highest M1 signature score, suggesting polarization towards the M1 phenotype (Figure S5H). Flow cytometry analysis exhibited that CXCL10 could downregulate the M2 macrophage marker genes (such as CD206 and CD163, Figure S5I). Notably, patients with a high proportion of TAM_C1_APOE had a shorter PFS, while the patients with a high proportion of TAM_C2_CXCL10 exhibited a longer PFS (Figure 5E and Figure S5J). The other two TAM clusters demonstrated transition phenotypes characterized by intermediate scores for both M1 and M2 signatures. Furthermore, we noted that after therapy, TAM_C3_FN1 displayed a significantly higher M1 score and a lower M2 score, whereas TAM_C2_CXCL10 showed a lower M2 score, and TAM_C1_APOE exhibited a higher M2 score, indicating evidence of TAM repolarization (Figure S5K). We found that non‐CR patients had decreased M1 signature scores and increased M2 signature scores, which indicated the M2 polarization from M1 of myeloid cells in these patients (Figure 5C). However, M1 polarization only happened in CR patients while the M1 signature score increased only in these patients. We found that the TAM_C2_CXCL10 population increased in patients with CR and decreased in those who did not (non‐CR), suggesting that these variations may contribute to the different responses to therapy observed in these two groups (Figure 5D and Figure S5B, C).

We then sent to probe whether the interferon produced by CD8^+^ T contributed to the TAMs evolution. IFN‐α and IFN‐γ response signature scores both increased in OT samples in CR patients but reduced in non‐CR patients (Figure 5F). Furtherly, patients with higher IFN‐α or IFN‐γ response signature scores had longer PFS (Figure S4L, M). In order to illuminate the temporal interaction between TAMs and other immune cells while on the treatment, we performed a cellular communication analysis. The results revealed that TAM_C2_CXCL10 had the most interaction strength with CD8^+^ T cells, which communicated with TAMs, especially TAM_C2_CXCL10 cells (Figure 5G). Nevertheless, we found that TAM_C2_CXCL10 cells can interact with CD8^+^ T cells via both IFNG‐IFNGR1/2 (Figure 5H, I) and CXCL10‐CXCR3 pairs (Figure 5J, K), respectively. As a marker gene of TAM_C2_CXCL10 cells, CXCL10 had a significant positive correlation with the fraction of TAM_C2_CXCL10 cells (Figure 5L). CXCR3 had a positive correlation with the fraction of CD8T_effector cells and a negative correlation with the fraction of CD8T_naive cells (Figure 5 M, N), which led us to guess that TAM_C2_CXCL10 cells could promote CD8^+^ T‐cell maturation. Also, CXCL10 could up‐regulated the expression of CXCR3 in CD8^+^ T cells, which indicated the interaction between TAM_C2_CXCL10 and CD8^+^ T cells (Figure S5N). In general, the abovementioned results indicated combinatory therapy triggers the shift to proinflammatory phenotype in TAMs. This shift may be directed through the close interaction with other immune cells, for example, CD8^+^ T cells, which attenuated via IFNG produced by CD8^+^ T cells, even if the TAMs exhibited higher expression of genes suppressing the inflammatory immune microenvironment which potentially promotes eventual resistance to the therapy. In turn, the TAM_C2_CXCL10 cells could activate the CD8^+^ T cells. All these changes respond to the different treatment efficacy of combination therapy.

### 3.6 Renovation of endothelial cells

We identified 672 endothelial cells and clustered them into four subpopulations (Figure S6A). EC_C1_SOX17 was identified as arterial ECs (SOX17, SEMA3G, and HEY1), while EC_C2_ACKR1 was venous ECs (ACKR1, NR2F2, and SELP), EC_C3_RGCC was general capillary ECs (RGCC, SPARC, and SGK1),^47^, ^48^, ^49^, ^50^, ^51^, ^52^, ^53^, ^54^ and EC_C4_S100A4 was a new aerocyte ECs cluster (RCSD1 and S100A4, but negative for other EC‐specific genes)^54^, ^55^ (Figure S6B). After the combination therapy, only patients with CR showed an increase in the proportion of EC_C4_ S100A4 cells evidenced by both scRNAseq data (Figure S6C) and bulk RNA‐seq samples therapy (Figure S6D). GO and KEGG signalling pathway enrichment analysis demonstrated that T‐cell differentiation, lymphocyte differentiation, and T‐cell activation signalling pathways were upregulated in EC_C4_CD27 cells, which exhibited the special immunologic function of this cluster in TME of NPC (Figure S6E, F).

To better comprehend the overall transcriptomic changes of endothelial cells (ECs) responding to the combination therapy, we conducted DEG and KEGG pathway enrichment analysis. Our findings revealed the upregulation of epithelial cell migration, tissue migration, endothelial cell differentiation signalling pathways, and regulation of angiogenesis in BT samples (Figure S6G). Conversely, genes upregulated in OT samples were associated with the positive regulation of cell adhesion, oxidative phosphorylation, aerobic respiration, and the negative regulation of cell motility. These findings collectively suggest a shift towards an anti‐tumour phenotype among ECs in OT samples, possibly triggered by anti‐angiogenesis therapy. Additionally, we noted a significant upregulation of oxidative phosphorylation signature and downregulation of angiogenesis both in non‐CR and CR patients (Figure S6H). However, the hypoxia signature increased in OT samples for non‐CR patients, whereas it decreased for CR patients. These results indicated that resistance to therapy emerged in a portion of patients, which may result from adapting to the hypoxic environment.

### 3.7 Heterogeneity of cancer cells

We further examined the malignant cells. A total of 1682 malignant cells were identified from the epithelial cell clusters which had a high level of EPCAM based on a high presence of chromosomal CNV compared with fibroblast cells as a reference (Figure S7A,B). There was a significant correlation of DEGs between BT versus OT comparison group and non‐CR versus CR patients of BT samples comparison group (*r* = .68, *p* < .001, Figure S7C). Genes related to Tregs recruiting in tumour (CCL20), tumorigenesis (SOX2, SOX4), and immune evasion (TPI1, SPP1) were upregulated in both comparison groups (Figure S7C).^56^, ^57^, ^58^, ^59^ SCENIC analysis revealed that FOS, MAZ, JUND, IRF7, TAF1, and the other TFs associated with signal transduction and tumour progression exhibited higher expression in BT group or non‐CR group (Figure S7D, E).^60^, ^61^, ^62^, ^63^ These results highlight substantial changes in transcriptomic profiles between malignant cells from before and after treatment, as well as between patients with non‐CR and those with CR. This suggests that combination therapy may halt the progression of malignant cells and induce alterations in certain molecular characteristics, potentially enhancing therapeutic efficacy.

**FIGURE 6:**
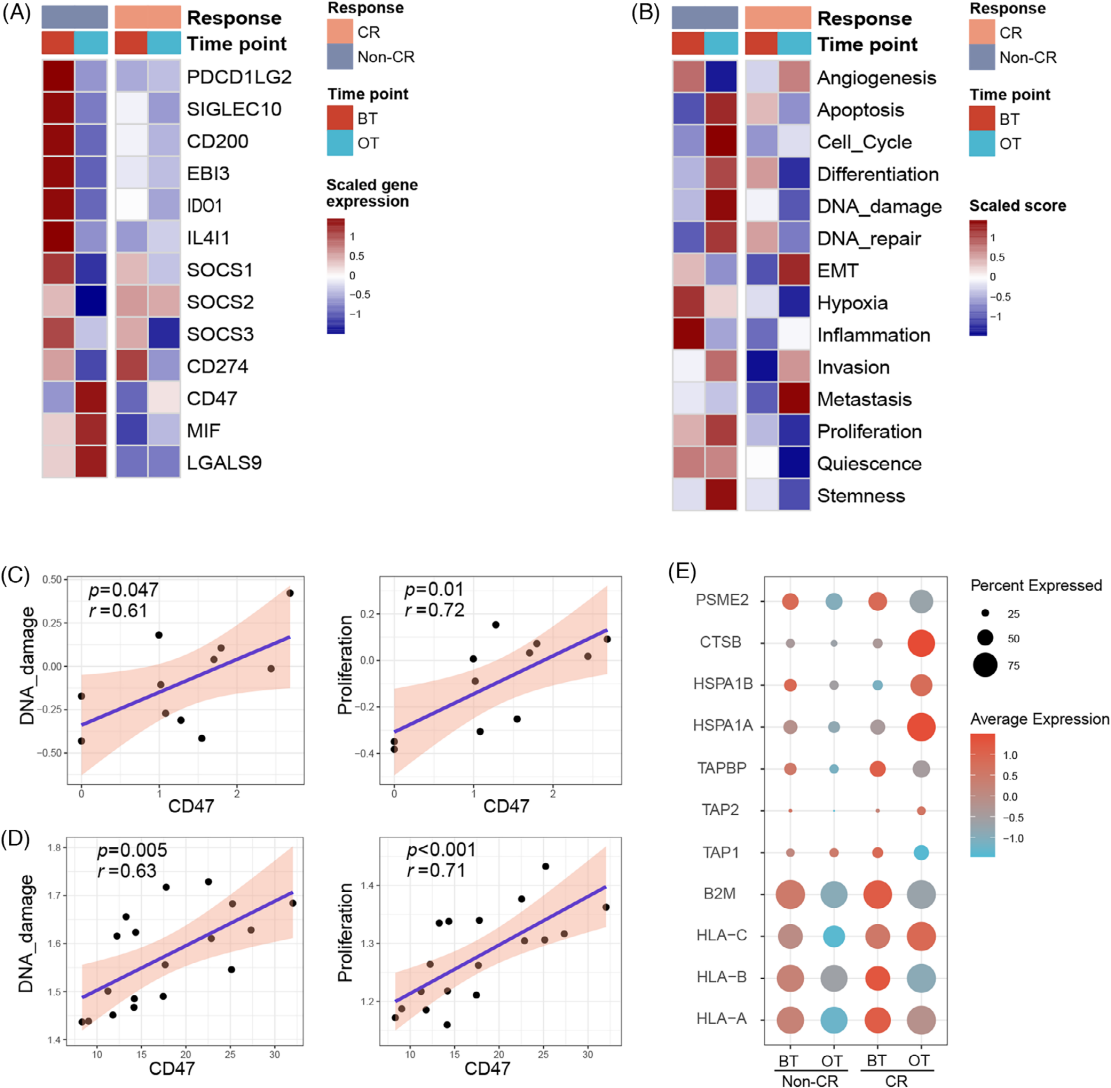
Malignant cells heterogeneity and changes response to therapy. (A) The heatmap shows the normalized mean expression of immune checkpoint genes and immune‐suppressive genes for comparisons of malignant cells between BT and OT samples for CR and non‐CR patients. (B) Heatmap shows the GSVA analysis using tumour microenvironment classification hallmark gene sets between BT and OT samples for CR and Non‐CR patients. (C) Plot of correlation between CD47 relative expression and DNA damage and proliferation signature score in malignant cells in the scRNA‐seq dataset. (D) Plot of correlation between CD47 relative expression and DNA damage and proliferation signature score in the bulk RNA‐seq dataset. (E) Dot plot shows the relative RNA expression of MHC‐I and antigen presentation genes in scRNA‐seq dataset. In A and B, solid colours from dark blue to dark red represent scaled gene expression levels and signature score from low to high, respectively.

We further explore the activity of immune checkpoint genes and immune‐suppressive genes, such as CD274 (PD‐L1), PDCD1LG2 (PD‐L2), SIGLEC10, SOCS1, SOCS2, SOCS3, CD200, EBI3, IDO1, IL4I1, and found that these genes generally reduced after therapy in all patients. Notably, non‐CR patients showed significantly higher levels of these genes in the baseline (BT) samples compared with CR patients, suggesting that lower expression of these markers may be associated with a better response to combination induction therapy (Figure 6A). Furthermore, genes expressed on malignant cells, such as CD47, MIF, and LGALS9, which mediate immunosuppressive effects, were also upregulated in BT samples of non‐CR patients compared with CR patients and were noticeably upregulated after therapy (Figure 6A). Similarly, GSVA analysis indicated that pathways related to angiogenesis, hypoxia, inflammation, proliferation, and quiescence were upregulated in non‐CR patients’ BT samples compared with CR patients (Figure 6B). Moreover, pathways related to apoptosis, differentiation, DNA damage, proliferation, and stemness were upregulated after therapy in non‐CR patients but downregulated in CR patients (Figure 6B). Furthermore, CD47 showed a significant positive correlation with DNA damage and proliferation signature both in scRNA sequencing and bulk RNA‐seq data (Figure 6C, D). Moreover, we observed that MHC‐I and antigen presentation genes were generally more highly expressed in CR patients in both BT and OT samples compared with non‐CR patients, and they were downregulated only in non‐CR patients after therapy (Figure 6E). These findings suggest that some patients have high levels of immune checkpoint genes at baseline which may be related to a poor response to the immunotherapy combined therapy, and they may adapt to immunotherapy by upregulating other immunosuppressive molecules (like CD47) or signature (like proliferation).

### 3.8 Intercellular interactions with the tumour microenvironment

**FIGURE 7:**
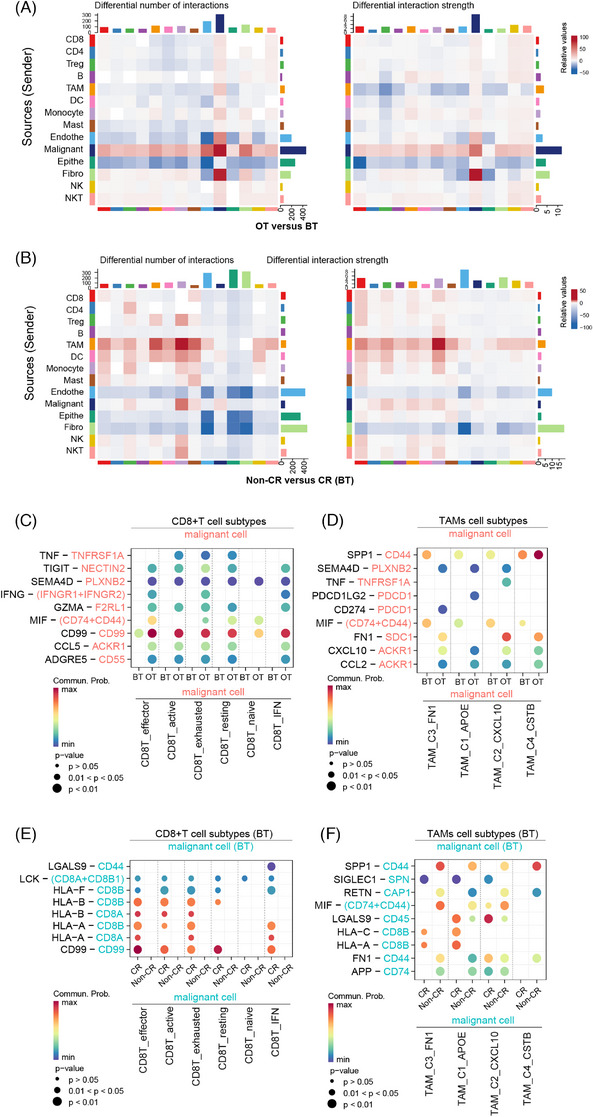
Complicated communication networks among subtypes. (A) Heatmap shows differential number of interactions or interaction strength in OT samples compared with BT samples. The top‐coloured bar plot represents the total incoming signalling across different cell types. The right coloured bar plot represents the total outcoming signalling across different cell types. In the coloured bar, red (or blue) represents increased (or decreased) signalling in OT samples compared with BT samples. (B) Heatmap shows differential number of interactions or interaction strength in non‐CR samples compared with CR samples for BT samples. The top‐coloured bar plot represents the total incoming signalling across different cell types. The right coloured bar plot represents the total outcoming signalling across different cell types. In the colour bared, red (or blue) represents increased (or decreased) signalling in non‐CR samples compared with CR samples. (C) Ligand–receptor interactions among CD8^+^ T‐cell subtypes and malignant cells between OT and BT samples. (D) Ligand–receptor interactions among TAMs cell subtypes and malignant cells between OT and BT samples. (E) Ligand–receptor interactions among CD8^+^ T‐cell subtypes and malignant cells between non‐CR and CR patients in BT samples. (F) Ligand–receptor interactions among TAMs cell subtypes and malignant cells between non‐CR and CR patients in BT samples. An interaction is indicated as colour‐filled circle at the cross of interacting cell types in a tissue(*x*‐axis) and a ligand–receptor pair (*y*‐axis), with circle size representing the significance of *p*‐value in a permutation test and colours representing the means of the communication proportion of the interacting pair.

We analyzed the changes in cellular communication associated with combination therapy and treatment response using the Cellchat.^25^ By calculating the interactions between different cell types stratified for a sample time point (BT versus OT) and response status (CR versus non‐CR), we found that cell–cell interactions number and intensity were both higher in OT group compared with BT samples (Figure S8A). Furthermore, CR patients showed more interactions than non‐CR patients in BT samples (Figure S8B), indicating that combination therapy resets complex cross‐talk among cell types, with CR patients exhibiting more interactions regardless of therapy exposure status. This suggests that such interactions could serve as markers predicting response to therapy. Consistent with these findings, we observed that malignant cells exhibited increased signalling to other cells (both in number and intensity) after therapy (Figure 7A). Conversely, interactions of TAMs with other cells decreased after therapy, although non‐CR patients showed more of these interactions than CR patients in BT samples (Figure 7B).

In addition to the abovementioned interactions between TAMs and CD8^+^ T cells, we focused on the interactions between malignant cells with either CD8^+^ T cells or TAMs. In the OT samples, we observed an increase in predicted interaction pairs between CD8^+^ T cells and malignant cells compared with BT samples. These pairs, such as TNF_TNFRSF1A, SEMA4D_PLXNB2, MIF_(CD74+CD44), CD99_CD99, CCL5_ACKR1, are crucial for immune regulation and tumour elimination (Figure 7C).^64^, ^65^, ^66^ Particularly noteworthy, the increase in interaction pairs involving IFNG_(IFNGR1+IFNGR2) in OT samples, indicating that the combination therapy enhances the secretion of interferon by CD8^+^ T cells, which in turn interact with malignant cells to execute anti‐tumour effect.^67^ In OT samples, most of the pairs between TAM cell subtypes and malignant cells enhanced compared with BT samples (Figure 7D). However, interactions involving TAMs signalling to malignant cells expressing CD44 via SPP1 and MIF, known to suppress T‐cell activation and promote TAM secretion of growth factors respectively,^68^, ^69^ decreased after therapy (Figure 7D), suggesting a transformation towards an anti‐tumour microenvironment.

In BT samples, the most frequent ligand–receptor pairs between CD8^+^ T cells to malignant cells were related to antigen presentation, including HLA_A/B/C/F_CD8A/B/C/F, HLA_E_CD8B, which were related to antigen presentation and CD8^+^ T‐cell activity. These pairs were more frequent in CR patients compared with those with non‐CR (Figure 7E),^70^ highlighting the importance of HLA expression before treatment in CD8^+^ T‐cell recognition and tumour cell killing, thereby the therapeutic effect. Similarly, antigen presentation ligand–receptor pairs, such as HLA_A/C_CD8B between TAM subtypes and malignant cells were more frequent in CR patients than non‐CR patients in BT samples (Figure 7F). Conversely, In non‐CR patients, TAMs cell subtypes were predicted to interact more interaction with malignant cells through SPP1_CD44, MIF_(CD74+CD44), which are known to be involved in immune‐suppressive (Figure 7F).^68^, ^69^ CD8^+^ T cells also had broad interactions with TAMs cells (Figure S8C, D). For instance, ANXA1_(FPR2+LXA4), ADGRE5_CD55, CXCL11_CXCR3, and CCL4_CCR5, most of which were related to immune regulation, were upregulated after therapy (Figure S8C).^71^, ^72^, ^73^ In BT samples, CD8T_exhausted cells were inferred to have a higher density of interaction with TAMs via CSF1_CSF1R signalling in non‐CR patients versus CR patients, of which a classic tumour‐promoting cytokine (Figure S8D).^74^ We also note that TAM_C2_CXCL10 had more interaction with CD8^+^ T cells in CR patients versus non‐CR patients of BT samples via JAM1_(ITGAL+ITGB2) signalling, which was associated with the process of leukocyte transmigration and tumour suppression (Figure S8D).^75^, ^76^ Taken together, these results exhibited an interactive immune environment following combined anti‐PD1 treatment and portrayed the different interactions between CR and non‐CR patients.

## 4 DISCUSSION

The intricate interplay among malignant cells, immune/stromal cells, and their interactions within the NPC TME critically influences clinical outcomes.^77^, ^78^ In this study, we utilized coupled scRNA‐seq and bulk RNA‐seq analyses of paired samples collected before treatment and during treatment from metastatic lymph nodes of NPC patients undergoing combination induction therapy, which represents the largest collection of NPC scRNA‐seq and bulk RNA‐seq data to date. This dataset encompasses treatment‐naïve and paired on‐treatment samples from patients receiving ICB combined induction therapy. Our approach allowed for an unbiased assessment of inter‐tumoral changes in NPC patients receiving the combination induction treatment and provided insights into how TME is affected by treatment and how baseline TME characteristics correlate with patient response. For the first time, we revealed a comprehensive regulation in TME upon the combination therapy in metastatic NPC.

Despite the considerable heterogeneity of NPC, we observed relatively clear genomic differences between samples collected before and during treatment, as well as baseline TME characteristics associated with patient response to cytotoxic therapy. Notably, we found that pro‐TME signatures were suppressed following treatment, and patients with CR exhibited milder features at baseline compared with patients with non‐CR. Conversely, anti‐TME signatures were widely activated evidenced in bulk RNA‐seq data after treatment. These findings offer a new insight into the potential for personalized early combination therapy targeting the TME.

Previous studies have reported that CD8^+^ T cells were the critical effector of killing tumour cells.^79^ In line with previous studies,^14^, ^80^ we observed widespread expression of both cytotoxic and exhausted markers among CD8^+^ T subtypes. It is reported that patients with elevated expression of exhaustion and cytotoxicity genes in CD8^+^ T cells potentially benefited from anti‐PD‐1 therapy.^81^ However, prevention or reversion of the CD8^+^ T cells’ exhaustion status and restoration of its cytotoxic status was critical for improving the ICB anti‐tumoral efficacy.^82^ In the present study, we revealed a reduced proportion of exhausted CD8^+^ T cells and increased active CD8^+^ T cells after therapy, which is consistent with the previous report.^83^ Intriguingly, we noted that the CD8^+^ T cells shift towards progenitor exhausted phenotype after therapy which could predict positive clinical outcomes as indicated in previous studies.^84^ Our study also showed that both exhaustion and cytotoxicity markers were upregulated in CR patients after therapy whereas downregulated in non‐CR patients, suggesting the potentiality of these markers reflecting the treatment efficacy in NPC patients.

Different from that myeloid cells increased after receiving GP‐inducing therapy in a recent study, patients in this study showed a decreased proportion of myeloid cells, which may be attributed to the different induced therapy with the combination regimen. TAMs which act as mediators of immunity are proven to perform a variety of roles with diverse phenotypes, including angiogenesis, metastasis, tumour growth, and inflammation, as well as tumour killing.^85^, ^86^ In the present study, checkpoint molecules (VSIR, VSIG4, LGALS9, SIGLEC10) expression in TAMs were activated after therapy, suggesting the adaptation of the immune system which may underlie treatment resistance.^41^, ^87^ It was reported that malignant cells can be phagocytosed by proinflammatory M1 macrophages, but can be promoted by anti‐inflammatory M2 macrophages.^88^ In this study, we identified a subtype of TAM_C2_CXCL10 and TAM_C1_APOE cells, which had M1 and M2 polarized, respectively. TAMs were proved to shift to proinflammation phenotype as the intermediate population cells expressed higher M1 signature after therapy. Furthermore, the fraction of the TAM_C2_CXCL10 cells increased in CR patients but decreased in non‐CR patients, which also be found positively associated with longer PFS in NPCs. Also, the IFN‐α and IFN‐γ response signatures were activated in CR patients but suppressed in non‐CR patients. This was also observed in CD8^+^ T cells. Besides, IFNG secreted by CD8^+^ T cells was higher in CR patients and decreased only in non‐CR patients after therapy. It was proved that CD8^+^ T cells interacted with TAMs, particularly in TAM_C2_CXCL10, via IFNG_IFNGR1/2 pairs. In turn, TAM_C2_CXCL10 interacted with CD8^+^ T cells via CXCL10_CXCR3 pairs, which would promote the maturation of CD8^+^ T cells. These observations suggested that the successful shift to proinflammatory TAMs was the intrinsic reason patients got better benefits from the combination therapy. This procession was driven by the interferon produced by CD8^+^ T cells and eventually promoted its maturation in turn. However, this process failed in the other patients.

The results also suggested potential molecular targets whose modulation could be synergistic with anti‐PD‐1 therapy. For instance, we found that IFN‐related genes were widely upregulated after therapy in CR patients, but downregulated in Non‐CR patients in both CD4^+^ T and CD8^+^ T cells. Also, TAMs in CR patients exhibited higher IFN response signatures after therapy, while it was suppressed in non‐CR patients. Previous studies have shown that IFN response can not only drive immune activation but also promote immunosuppressive effects, which mainly depend on tumour burden and composition of TME.^89^, ^90^ Therefore, based on the findings above, we infer the hypothesis that the combination therapy enhanced the tumour‐killing effect by reversing the exhaustion of CD8^+^ T cells and increasing the proportion of other cytotoxic CD8^+^ T cells. Ulteriorly, CR patients expressed more fraction of CD8T_IFN cells than non‐CR patients, and higher levels of IFN‐related genes, which act on TAMs and induce diverse transformation. All these differences respond to their ultimate outcomes, and combining interferon therapy would be a promising strategy for NPC patients’ therapy which had been verified in various other cancers previously.^91^, ^92^ Similarly, we observed suppressive interactions between CD8^+^ T and TAMs, malignant cells, and TAMs (for example, SPP1‐CD44, MIF‐[CD74+CD44]), which varied between treatment exposure and treatment efficacy and potential be immunoregulatory targets. Detailed mechanistic studies are wanted to validate this putative model and help explain the different changes of the TME between patients during treatment. Besides, our study revealed that patients with relatively worse efficacy have higher levels of immune checkpoint molecules before therapy, and they may adapt to immunotherapy by upregulating other immunosuppressive molecules and decreasing the presentation of tumour antigens. Thus, patients with high levels of immune checkpoint molecules before treatment may be better treated by a combination of, for example, anti‐TIM or anti‐CD47 drugs, which have also been explored in previous studies.^15^, ^93^, ^94^ Detailed mechanistic studies are warranted to validate this putative model and help explain the different changes of the TME between patients during treatment.

We acknowledge several limitations in our study. Firstly, we only included metastatic lymph node biopsy samples, which were still relatively small in size, for both scRNA‐seq and bulk RNA‐seq analysis. To validate our findings, a large cohort including samples from both nasopharyngeal and distant metastatic sites is needed. Second, the uniformity of the treatment among enrolled patients may limit the generalizability of our findings, and comparison with other treatment strategies is necessary to confirm some of our observations. Third, additional analysis such as TCR/BCR profiling, single‐cell proteomics, and spatial transcriptomics are needed to further elucidate the complexity of TME. Lastly, fundamental experiments are required to explore the underlying mechanisms of our findings. Currently, we are expanding our sample size by including samples from different metastatic sites and with varying therapy strategies using TCR/BCR scRNA‐seq analysis.

In summary, our study provides initial and validation datasets of pretreatment and paired on‐treatment NPCs, which contextualize novel combination approaches being clinically tested. We have generated a detailed TME map across different treatment exposure statuses and response categories, offering insights into mechanisms underlying therapy response and facilitating the development of individualized and effective immunotherapy strategies.

## AUTHOR CONTRIBUTIONS

Yaofei Jiang, Guoying Liu, Yanqun Xiang, and Changqing Xie conceived the study. Yaofei Jiang, Weixin Bei, Wangzhong Li, Ying Huang, and Shuiqing He collected and prepared the samples. Ying Huang, Changqing Xie, Xiaobin Zhu, Lisheng Zheng, Weixiong Xia, Shuhui Dong, Qin Liu, Chuanrun Zhang, and Shuhui Lv collected the data and performed the methodology. Yaofei Jiang, Weixin Bei, Wangzhong Li, and Xiaobin Zhu performed the statistical analyses. Yaofei Jiang, Weixin Bei, Wangzhong Li, Changqing Xie, Yanqun Xiang, and Guoying Liu interpreted the data. Yaofei Jiang, Changqing Xie, Yanqun Xiang, and Guoying Liu wrote the original draft. All authors read and approved the final manuscript.

## CONFLICT OF INTEREST STATEMENT

The authors declare no conflict of interest.

## ETHICS STATEMENT

This study is an exploratory result of our previously published clinical trial and was approved by the Chinese Ethics Committee of Registering Clinical Trials (ChiCTR2000032317). All enrolled patients were informed and provided written consent.

## CONSENT FOR PUBLICATION

Not applicable.

## Supporting information



Supporting Information

Supporting Information

Supporting Information

Supporting Information

Supporting Information

Supporting Information

Supporting Information

Supporting Information

Supporting Information

Supporting Information

Supporting Information

Supporting Information

Supporting Information

## Data Availability

The raw data that support the findings of this study are available from the corresponding author upon reasonable request. Also, the scRNA‐seq and bulk‐seq processed data have been deposited in Zenodo (https://doi.org/10.5281/zenodo.13736891).
