# Supplementary material for: Single‐cell transcriptome analysis reveals evolving tumour microenvironment induced by immunochemotherapy in nasopharyngeal carcinoma
Source: Clin Transl Med. 2024 Oct 16;14(10):e70061. doi: 10.1002/ctm2.70061 (PMC11483602; doi:10.1002/ctm2.70061)
Supplement: Supplementary file 1 — Supporting Information [file CTM2-14-e70061-s002.docx]

**Supplementary Figure Legend:**

**FIGURE S1 Basic information of the single cell RNA sequencing. (A)** Histogram shows the number of single and doublet cells in each sample evaluated by “DoubletFinder” R package. The samples were named as following example, “BT2” and “OT2” representing before and on treatment metastatic cervical lymph nodes of patient P2, respectively. (**B)** UMAP of all high-quality cells, as indicated by the labels. Red blood cells were indicated in red which were removed for the following analysis. (**C)** Violin plots show the number of genes, number of unique molecular identifier (UMI), and proportion of mitochondrial gene counts of initial clusters. (**D)** Violin plots show the RBC related genes expression across the initial clusters. (**E)** Boxplots exhibits the number of UMI and genes in each major cell types. (**F)** Boxplots exhibits the number of UMI and genes in each sample.

**FIGURE S2. Major cell types basic information and T/NK subtypes identified. (A)** Bar plots of the composition proportion of the major cell types, which was normalized to the total cells; bar plot of the composition proportion of the CD8^+^ T cell subtypes, which was normalized to all CD8^+^ T cells; and bar plot of the composition proportion of the CD4+ T cell subtypes, which was normalized to all CD4^+^ T cells. (**B)** Boxplots illustrates the fraction of the major cell types in 18 paired BT and OT bulk RNA-seq samples, which were inferred by CIBERSORTx. Significance of differential proportion (p value) was determined by paired t-test. (**C)** Progression-free survival curves of the patients with NPC from GSE102349 dataset, stratified by the fraction of epithelial cells and endothelial cells which were inferred by CIBERSORTx. The red line shows the survival curve of the patients contain high cell fraction, while the blue line shows the survival curve of the remaining patients. (**D)** Violin plots show the number of genes, number of UMI, and proportion of mitochondrial gene counts of T cell clusters. (**E)** Proportions of the T cell subtypes in paired BT and OT samples. Significance of differential proportion (*p* value) was determined by paired t-test. (**F)** Violin plots show the naive signature scores, cytotoxic signature score, and exhausted signature score of each T cell clusters.

**FIGURE S3.** Immunohistochemical staining with LYZ, CD68, and MS4A6A in BT and OT samples. Scale bar, 100 μm.

**FIGURE S4.** **Expression profiles of CD8^+^ T, CD4^+^ T and Treg cells. (A)** Signature score distributions for progenitor signatures within exhausted CD8^+^ T cells between BT and OT samples. (**B)** Progression-free survival curves of the patients with NPC from GSE102349 dataset, stratified by progenitor signature score. The red line shows the survival curve of the patients contain higher progenitor signature score, while the blue line shows the survival curve of the remaining patients. (**C)** Bubble plot shows the KEGG analysis of DEGs for CD8T_exhausted cells between OT versus BT samples. (**D**) ELISA assay detected the expression of GZMK and GZMA in serum of the three CR and three Non-CR patients. (**E**) Development trajectory of CD4^+^ T cells derived from EBV DNA BT and OT samples. Cells were colored by cell states. (**F**) Radiation heatmap shows the differential genes expression patterns along the transformation of CD4^+^ T cell fate (**G)** GSEA of DEGs for Treg cells from OT samples compared with BT samples. (**H)** Heatmap shows the normalized mean expression of genes related to IL2R receptors, inhibitory molecules, co-stimulatory molecules and chemokines in Treg cell sub-clusters. Filled color from dark blue to dark red represent scaled expression levels from low to high. (**I)** Bubble plot shows the GO analysis of DEGs between Treg cell sub-clusters. (**J)** Bubble plot shows the KEGG analysis of DEGs between Treg cell sub-clusters. (**K)** Boxplots show the fraction of the Treg_suppressive cells in 18 paired BT and OT bulk RNA-seq samples, which were inferred by CIBERSORTx. Significance of differential proportion (p value) was determined by paired t-test. (**L)** Progression-free survival curves of the patients with NPC from GSE102349 dataset, stratified by the fraction of Treg_suppressive cells which were inferred by CIBERSORTx. The red line shows the survival curve of the patients contain high cell fraction, while the blue line shows the survival curve of the remaining patients. In A, significance of differential signature enrichment (p value) between samples was determined by t-test. ns: not significant. * p < 0.05, ** *p* < 0.01, *** *p* < 0.001, **** *p* < 0.0001.

**FIGURE S5.** **Characteristics of TAMs and DC cells. (A)** Differential gene expression analysis showing up- and down-regulated genes across all ten myeloid clusters. Genes up regulated with an adjusted *p* value < 0.01 is indicated in red, while genes down regulated with an adjusted *p* value < 0.01 is indicated in blue. Genes with an adjusted *p* value ≥ 0.01 is indicated in black. (**B)** Bar plot of the composition proportion of different myeloid subtypes. The proportion was normalized to all myeloid cells. (**C)** Proportion of the ten myeloid cell subtypes in paired BT (n=5) and OT (n=5) samples. Significance of differential proportion (p value) was determined by paired t-test. (**D)** The relative RNA expression of immunomodulatory genes in scRNA-seq dataset between OT and BT samples. (**E)** Volcano plot shows DEGs in TAM cells comparing OT with BT samples. (**F)** Volcano plot shows DEGs in TAM cells of BT samples, comparing CR with Non-CR samples. (**G)** Progression-free survival curves of the patients with NPC from GSE102349 dataset, stratified by the SPP1 expression. The red line shows the survival curve of the patients contain higher SPP1 expression, while the blue line shows the survival curve of the remaining patients. (**H)** Signature score distributions for M1-associated and M2-associated signatures between each TAM subtype. (**I)** Progression-free survival curves of the patients with NPC from GSE102349 dataset, stratified by the fraction of TAM_C1_APOE cell which were inferred by CIBERSORTx. The red line shows the survival curve of the patients contain high cell fraction, while the blue line shows the survival curve of the remaining patients. (**J**) Flow cytometry analysis detected the expression of CD163 and CD206 in THP-1 cells treated with recombinant CXCL10. (**K)** Signature score distributions for M1-associated and M2-associated signatures between BT and OT samples in each TAM subtype. (**L)** Progression-free survival curves of the patients with NPC from GSE102349 dataset, stratified by the IFN-α response signature score. (**M)** Progression-free survival curves of the patients with NPC from GSE102349 dataset, stratified by the IFN-γ response signature score. The red line shows the survival curve of the patients contain higher IFN-α or IFN-γ response signature score, while the blue line shows the survival curve of the corresponding remaining patients. (**N**) Flow cytometry analysis detected the expression of CXCR3 in CD8T cell treated with recombinant CXCL10. In D, H, and J, significance of difference (p value) between groups was determined by t-test. ns: not significant. * *p* < 0.05, ** *p* < 0.01, *** *p* < 0.001, **** *p* < 0.0001.

**FIGURE S6. Endothelial cells swing to anti-tumor phenotype after therapy. (A)** UMAP of endothelial sub-cluster cells, as indicated by the colors and labels. (**B)** Bubble plot shows the expression of cluster-specific marker genes between endothelial sub-cluster cells. (**C)** Bar plot of the composition proportion of different endothelial subtypes. The proportion was normalized to the total endothelial cell. (**D)** Boxplot showing the fraction of the EC_C4_CD27 cells in 18 paired BT and OT bulk RNA-seq samples, which were inferred by CIBERSORTx. Significance of differential proportion (p value) was determined by paired t-test. (**E)** Bubble plot shows the Gene Ontology (GO) analysis of DEGs between endothelial sub-clusters. (**F)** Bubble plot shows the Kyoto Encyclopedia of Genes and Genomes (KEGG) analysis of DEGs between endothelial sub-clusters. (**G)** KEGG analysis for up regulated DEGs in BT samples comparing to OT samples; and in OT samples comparing to BT samples. (**H)** Signature score distributions for oxidative phosphorylation, hypoxia and differentiation signatures in endothelial cells between BT and OT samples for CR and Non-CR patients. Significance of differential signature enrichment (p value) between samples was determined by t-test. ns: not significant. * *p* < 0.05, ** *p* < 0.01, *** *p* < 0.001, **** *p* < 0.0001.

**FIGURE S7. Single-cell copy-number variation (CNV) and malignant cells profiles between treatment status and responses. (A)** Heatmap showed the large-scale CNVs of all epithelial cells which was inferred based on scRNA-seq data. (**B)** K-means clustering analysis was performed on all epithelial cell clusters. Violin plot showed the CNV scores for each cluster derived from K-means clustering. Epithelial cells in the cluster (cluster 4) that predominantly contained fibroblasts cells were regarded as non-malignant cells. In contrast, the cells with high CNV scores in other clusters were identified as malignant cells. (**C)** Scatterplot comparing NPC gene expression fold changes from BT versus OT analysis (x axis) against fold changes from Non-CR versus CR analysis (y axis). DEGs were identified using “FindMarkers” R package base on default set and only those fold change >1 or < -1 for either comparison was shown. Red dots, genes upregulated in both comparison group; Blue dots, genes downregulated in both comparison group; Black dots, genes upregulated in either one comparison group. (**D)** Heatmap showing the activity of transcription factors (TFs) in malignant cells derived from BT versus OT samples. (**E)** Heatmap showing the activity of transcription factors (TFs) in malignant cells derived from Non-CR versus CR patients in BT samples.

**FIGURE S8. Interaction number, strength, and pairs between subtypes.** **(A)** The histography showing the number of interactions and interaction strength in BT and OT samples. (**B)** The histography showing the number of interactions and interaction strength in CR and Non-CR samples for BT samples. (**C)** Selected ligand-receptor interactions among CD8^+^ T cell subtypes and TAMs cell subtypes between OT and BT samples. (**D)** Selected ligand-receptor interactions among CD8^+^ T cell subtypes and TAMs cell subtypes between Non-CR and CR patients in BT samples. An interaction is indicated as color-filled circle at the cross of interacting cell types in a tissue(x-axis) and a ligand-receptor pair (y-axis), with circle size representing the significance of p value in a permutation test and colors representing the means of the communication proportion of the interacting pair.
