# Supplementary figures and images for: Single‐cell transcriptome analysis reveals evolving tumour microenvironment induced by immunochemotherapy in nasopharyngeal carcinoma
Source: Clin Transl Med. 2024 Oct 16;14(10):e70061. doi: 10.1002/ctm2.70061 (PMC11483602; doi:10.1002/ctm2.70061)

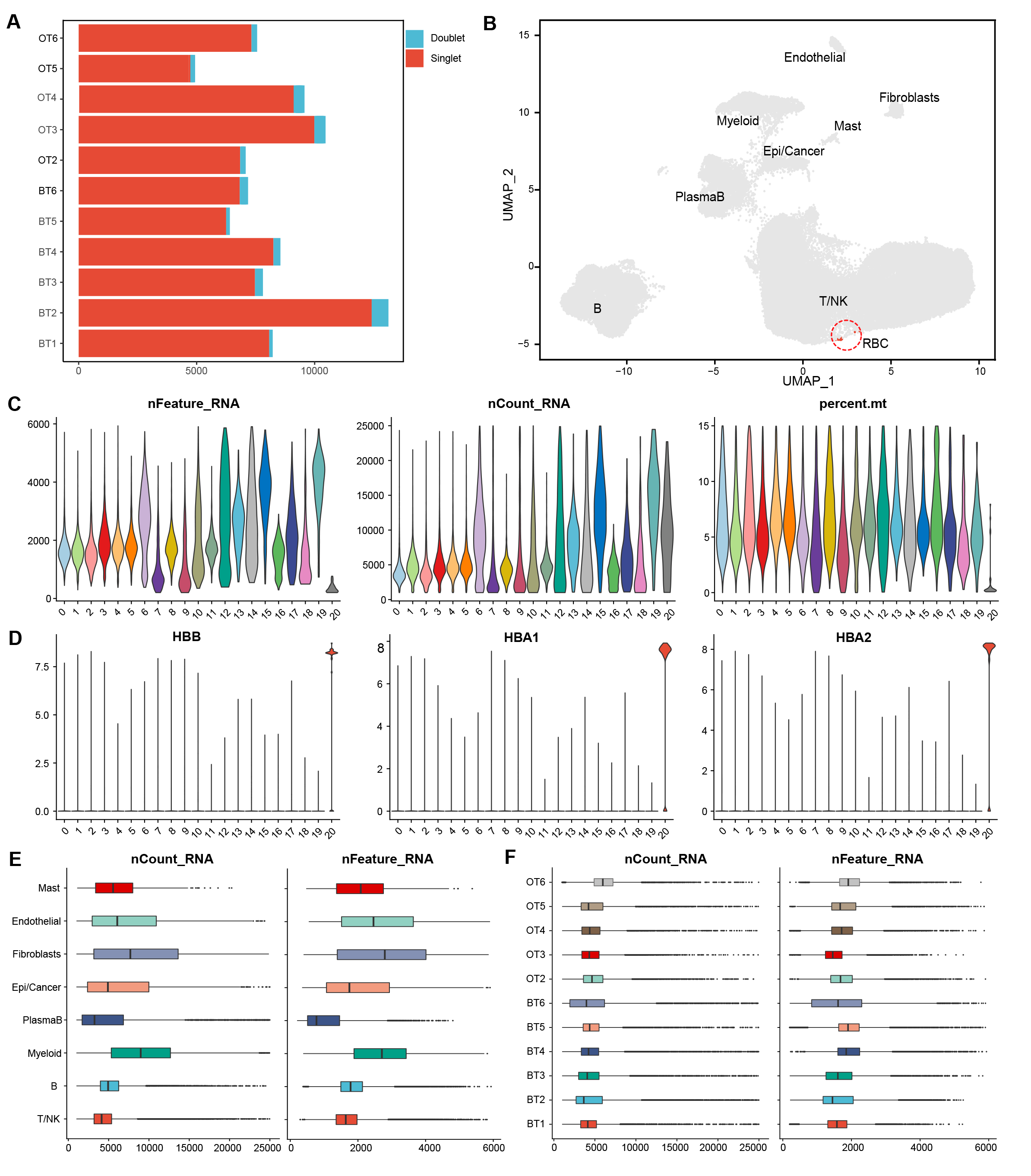

Supplement: Supplementary file 2 — Supporting Information [file CTM2-14-e70061-s003.tif]

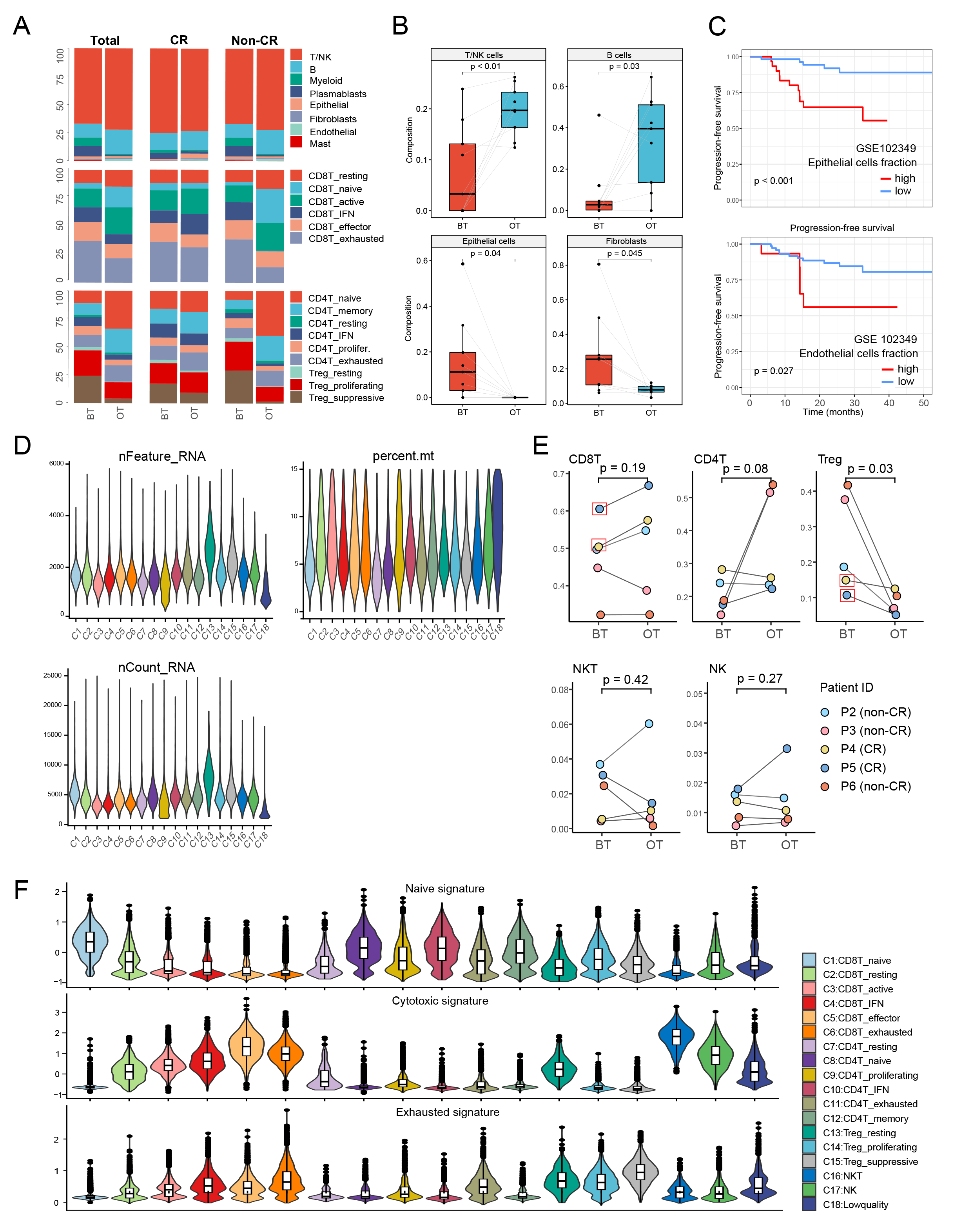

Supplement: Supplementary file 3 — Supporting Information [file CTM2-14-e70061-s013.tif]

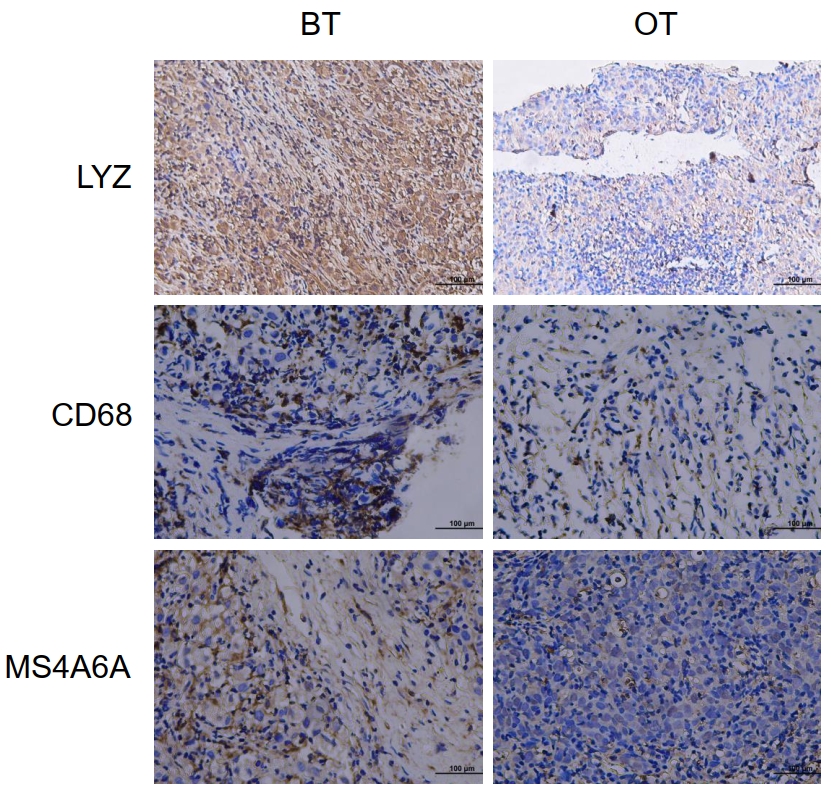

Supplement: Supplementary file 4 — Supporting Information [file CTM2-14-e70061-s009.tif]

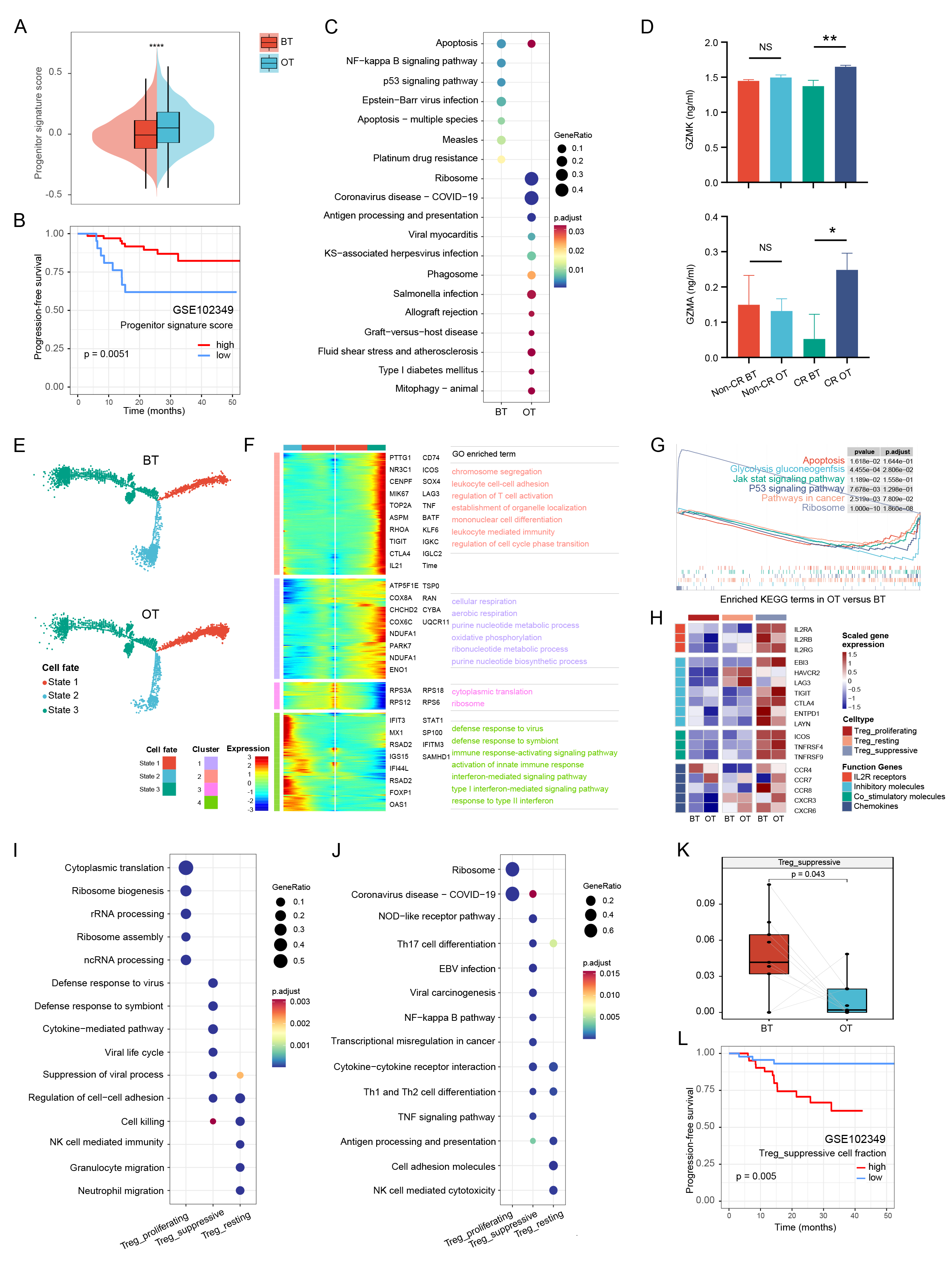

Supplement: Supplementary file 5 — Supporting Information [file CTM2-14-e70061-s010.tif]

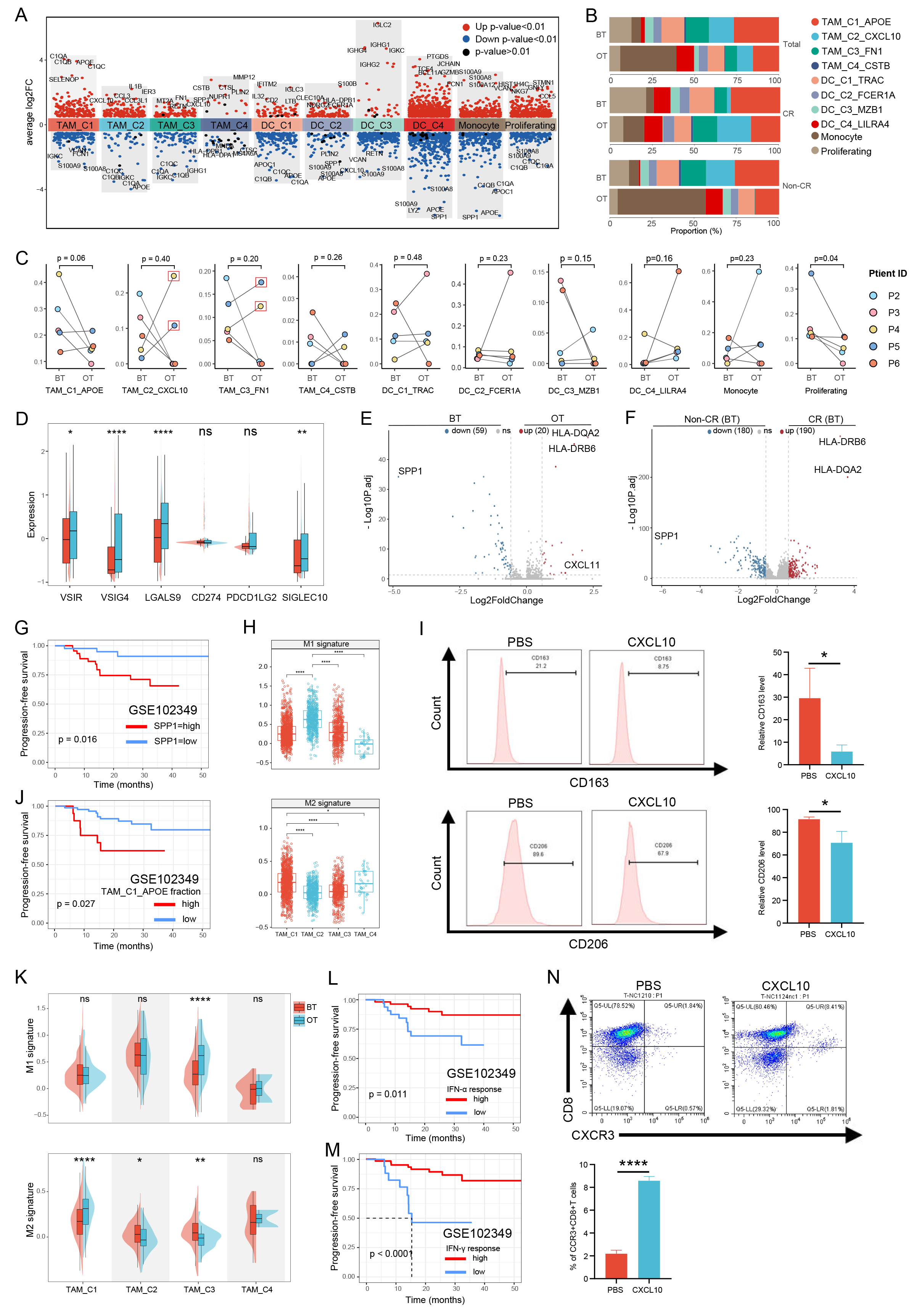

Supplement: Supplementary file 6 — Supporting Information [file CTM2-14-e70061-s007.tif]

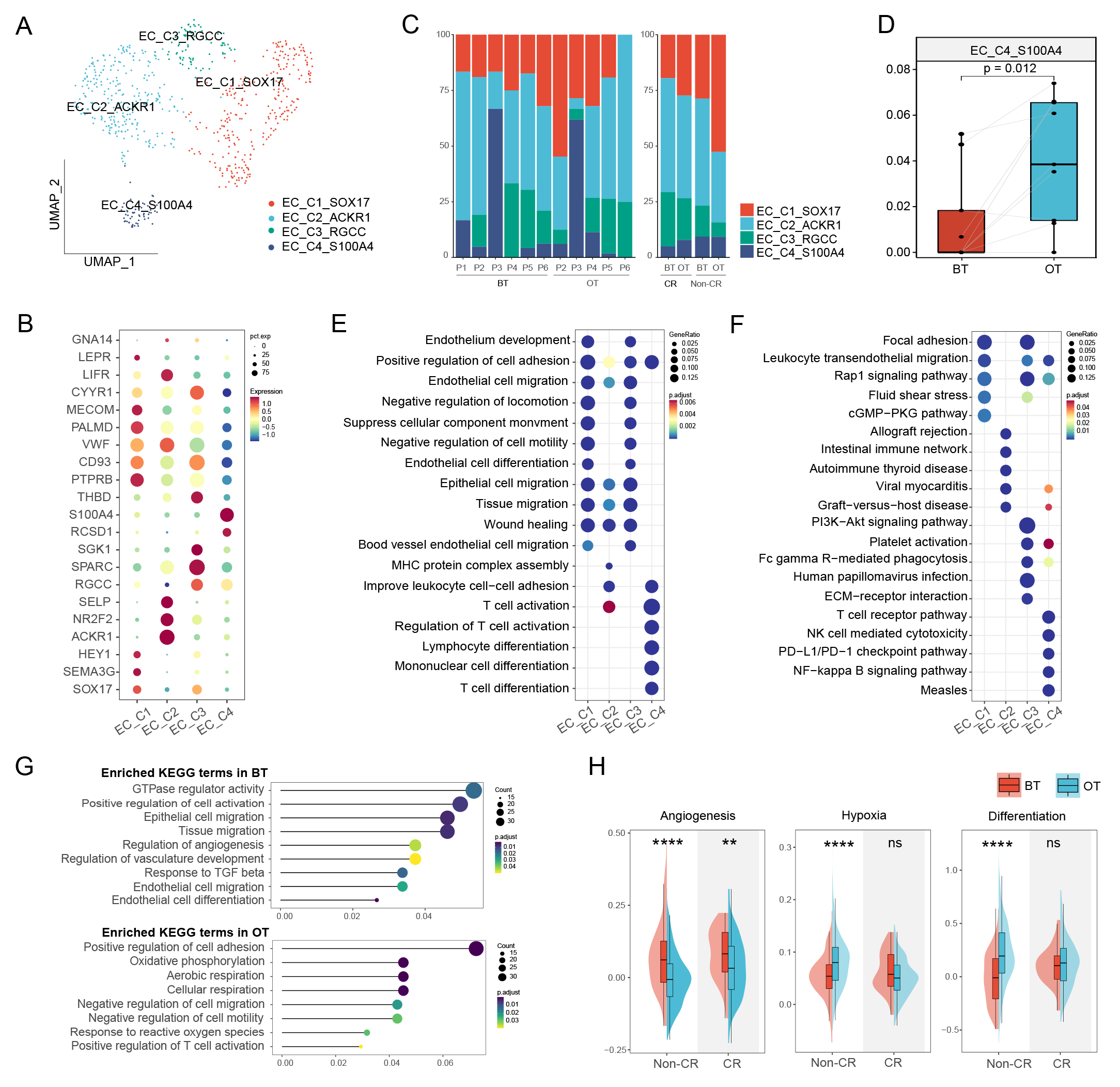

Supplement: Supplementary file 7 — Supporting Information [file CTM2-14-e70061-s011.tif]

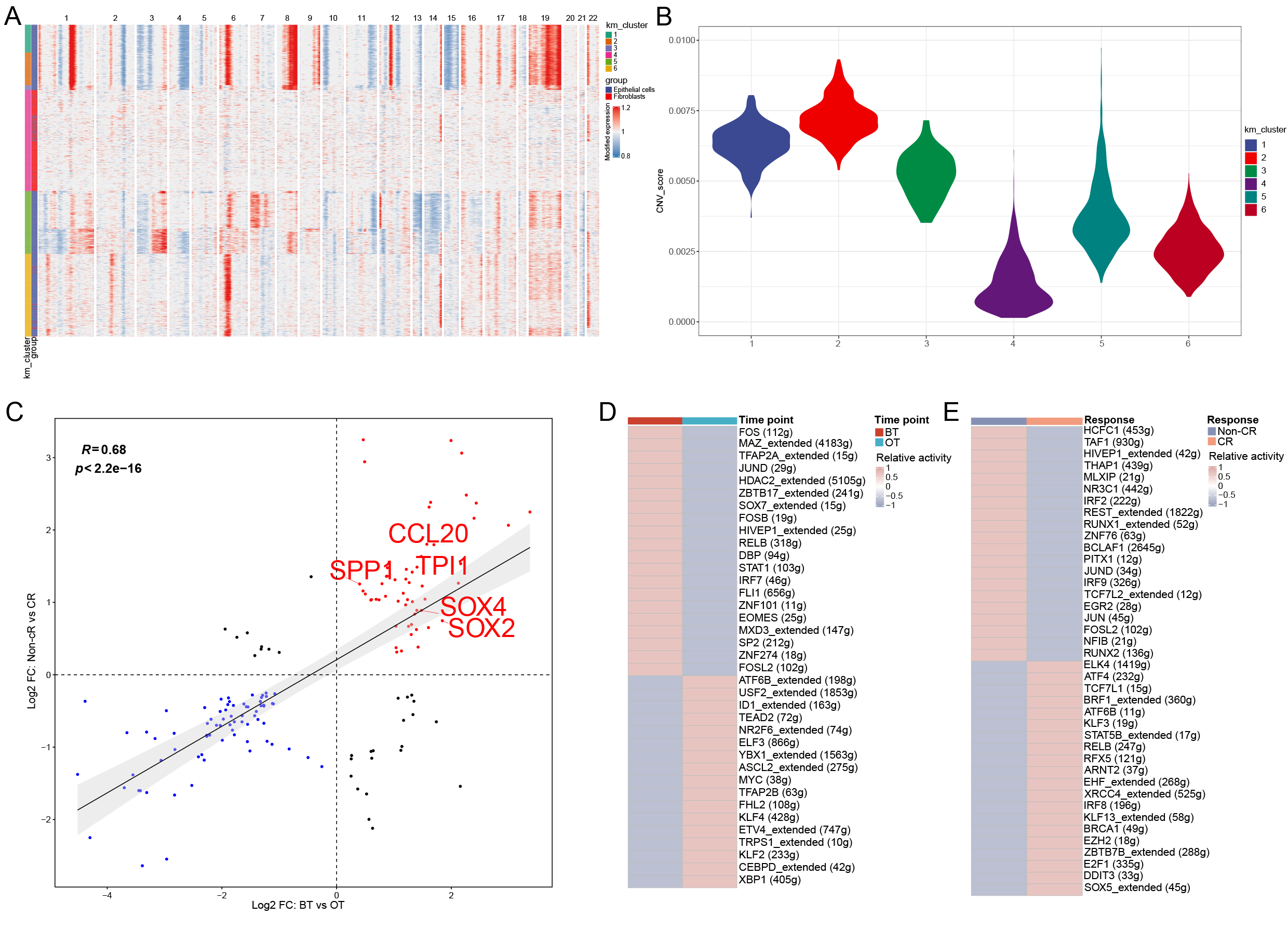

Supplement: Supplementary file 8 — Supporting Information [file CTM2-14-e70061-s004.tif]

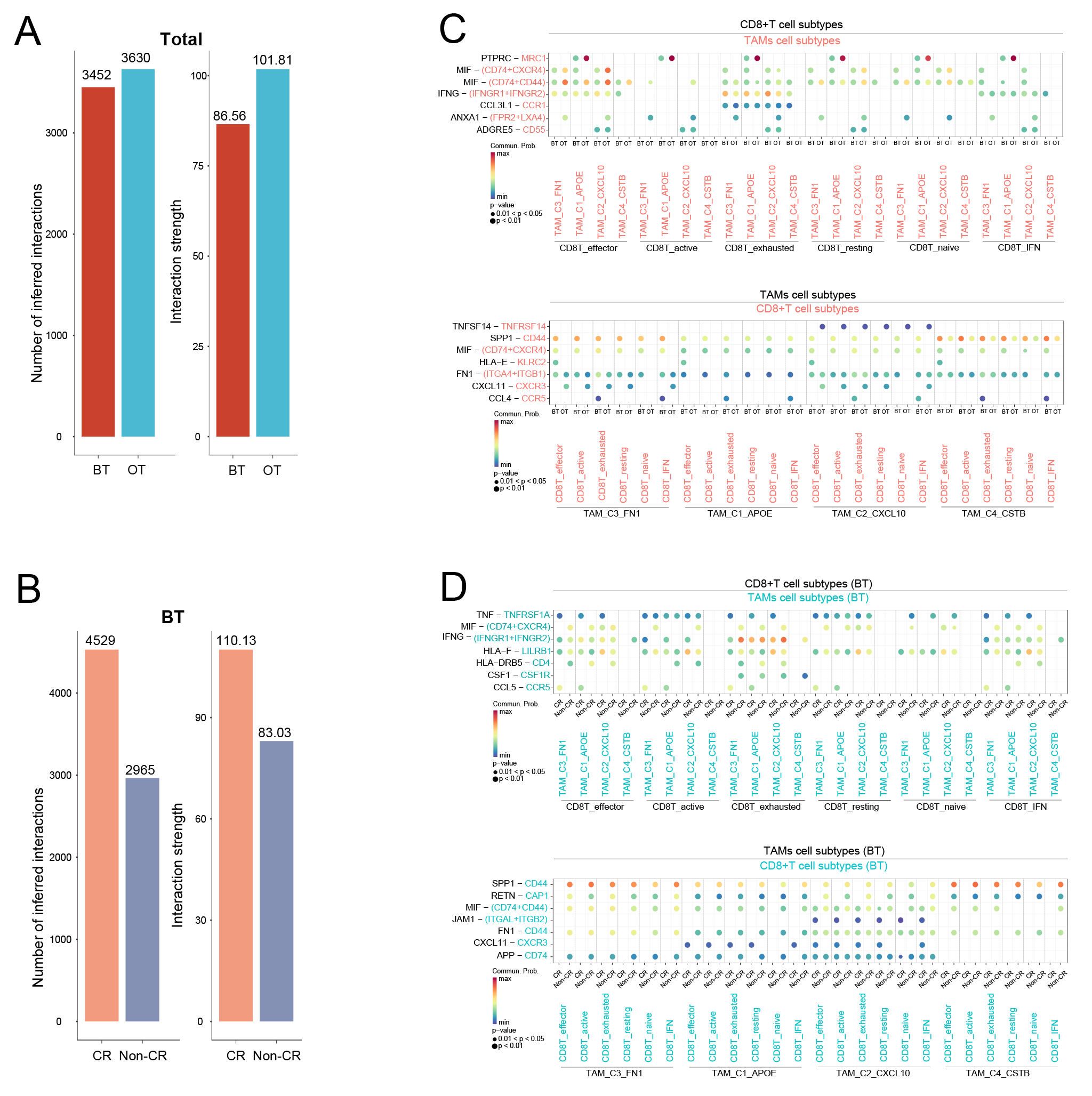

Supplement: Supplementary file 9 — Supporting Information [file CTM2-14-e70061-s012.tif]
